# On the (limited) use of touchscreen-based behavioural and cognitive research with dogs: potential causes and future directions

**DOI:** 10.1007/s10071-026-02055-3

**Published:** 2026-05-25

**Authors:** Siqi Yang-Fu, Christian Menne, Chiara Canori, Dániel Rivas-Blanco, Oli Green, Friederike Range, Tiago Monteiro

**Affiliations:** 1 Domestication Lab, Department of Interdisciplinary Life Sciences, Konrad Lorenz Institute of Ethology, University of Veterinary Medicine Vienna, Vienna, Austria; 2 Department of Medicine and Surgery, University of Parma, Parma, Italy; 3 William James Center for Research, University of Aveiro, Aveiro, Portugal; 4 Centre for Research in Animal Behaviour, University of Exeter, Exeter, UK

**Keywords:** Automated testing systems, Behaviour, Comparative Cognition, Canine-Computer Interaction, Dog, Touchscreen

## Abstract

Dogs (*Canis familiaris*) are an increasingly popular model in comparative cognition research. Traditional research paradigms often rely on heavy experimenter involvement, which can introduce biases and inconsistencies. Touchscreen-based automated systems offer a solution by enhancing standardization, reducing experimenter effects, and improving data quality, and have been widely adopted for research with diverse species. However, their adoption in dog research has remained underexplored. This systematic review quantified dog behaviour and cognition literature in relation to touchscreen-based methodologies and evaluated existing touchscreen-based approaches. Our search confirmed that while the field of dog cognition continues to grow, touchscreen-based studies are exceptionally rare, with only fourteen such publications identified for the entire indexed record. We explored the potential reasons for this limited adoption by categorizing them into three main barriers: technical, such as the need for interdisciplinary skills; practical, including high costs and lengthy training requirements and *Umwelt*, relating to the dogs’ species-specific constraints. Finally, we propose strategies to overcome these obstacles, including the use of open-source solutions, establishing multi-lab collaborations, and designing interfaces better aligned with dogs’ *Umwelt*. We conclude that despite being underutilized, touchscreen-based methods hold significant promise for advancing canine research. By addressing current challenges through collaborative, open, and dog-centred practices, the field can integrate touchscreen-based methods more widely, enhance reproducibility, accelerate discovery, and obtain objective indicators of dogs’ behavioural and cognitive capabilities.

**Supplementary Information:**

The online version contains supplementary material available at 10.1007/s10071-026-02055-3.

## Introduction

Dogs (*Canis familiaris*) have become a favoured model in comparative research (Aria et al. 2021; Bensky et al. 2013). A bibliometric analysis showed an increase in publication of dog behavioural and cognitive articles since 2000, with an accelerating trend from 2010 onwards that was considerably steeper than the growth observed in animal cognition and behaviour research in general (Aria et al. 2021). Several factors might have contributed to this increase, including dogs’ accessibility as companion animals, their trainability, and the relatively low cost of recruiting pet dogs compared to maintaining laboratory colonies, making them an attractive and practical model for cognitive and behavioural research (Elliott et al. 2017; L. S. Hall and Boxall 2024).

Traditionally, research on dog behaviour and cognition has relied heavily on paradigms that require extensive involvement from a human experimenter. In these tasks, the experimenter presents stimuli, initiates trials, records the dogs’ choices and then dispenses (or withholds) rewards. For example, working-memory paradigms, require the experimenters to place a treat, impose a delay, and later invite the dog to engage (Craig et al. 2012; Fiset et al. 2003; Head et al. 1995; Krichbaum et al. 2021; H. C. Miller et al. 2009). Similarly, discrimination tasks depend on people to arrange and present various physical or sensory cues (e.g., colours, shapes, quantities, odours, or sounds), and record dogs’ choices by hand, either live through an external observer or retrospectively via video (Miletto Petrazzini and Wynne 2016; Milgram 2003; Milgram et al. 1994; Mongillo et al. 2017; Rivas-Blanco et al. 2024; Tanaka et al. 2000; Tapp et al. 2003). Reward contingencies (e.g., reinforcing correct responses and withholding or changing rewards for incorrect responses) are also frequently manually controlled (Ashton and De Lillo 2011; Handley et al. 2023; Milgram 2003; Yin et al. 2008).

Reliance on human operators, however, introduces potential inconsistencies and biases. Inconsistencies in stimulus placement, inter-trial intervals or reward delivery delays occur across trials, days, and experimenters, and can potentially impact dog performance. Measurement and judgement biases can arise when observers code behaviour differently across sessions or individuals, expectancy bias can lead experimenters to (consciously or unconsciously) signal desired responses, observer biases may cause selective observation or misinterpretation of behaviour, and inter-rater reliability problems can further inflate variance (Clark et al. 2020; Holzbach 1978; Johnen et al. 2017; Reynolds et al. 2021; Schmidt and Hunter 1996; Tuyttens et al. 2014).

Recognising these issues, researchers have launched multi-lab collaborations across a variety of model species, such as the ‘ManyDogs’ project (ManyDogset al. 2021) aiming to reduce variation across different research sites by coordinating methodologies while increasing statistical power through larger combined sample sizes (Alessandroni et al. 2025). These initiatives mark an important progress in addressing replication concerns by increasing the number of subjects and replications, which can help buffer site-specific deviations and can even out local inconsistencies. However, such large-scale collaborations are not feasible for all research questions or lab infrastructures. For example, studies requiring specialised, high-cost equipment, such as fMRI (Karl et al. 2020; Prichard et al. 2021), are not accessible or affordable for many laboratories.

Alternatively, many researchers have begun adopting technology-enhanced approaches to mitigate methodological inconsistencies and potential biases. For example, markerless video-based tools such as DeepLabCut or SLEAP have been used to automate pose extraction and behaviour annotation, therefore reducing the need for manual scoring and enhancing consistency across sessions and observers (Lauer et al. 2022; Mathis et al. 2018; Nath et al. 2019; Pereira et al. 2022). App-based platforms like ZooMonitor allow digitalised data-entry, enabling automated reliability tests to check observer consistency (Wark et al. 2019). There have also been monitor-based studies where presentation of the stimuli is standardised, sometimes combined with eye-tracking technologies (e.g., Barber et al. 2016; Mongillo et al. 2021; Somppi et al. 2012; Völter et al. 2020). These tools provide more standardisation over manual procedures, and each have distinct strengths, but in terms of automation, they typically target individual components of the study (standardisation of trial contingency, presentation of stimulus, detecting or recording of behaviour) rather than integrating the entire process in a closed-loop fashion.

Touchscreen-based systems, however, represent a more comprehensive approach: they unify stimulus control, standardised task control, direct response logging, automated reward delivery, and trial-wise data logging inside a single apparatus, supporting cross-site standardisation and rapid parameterisation of tasks (Dumont et al. 2020; Egelkamp and Ross 2019; Kangas and Bergman 2017; Orphanides and Nam 2017; Seitz et al. 2021). The closed loop between the stimuli presentation and animals’ responses enables animals to receive immediate feedback based on their interactions with the touchscreen, which in turn guides their behaviour (Ajuwon et al. 2023, 2024a, b; Kane et al. 2020; Lopes and Monteiro 2021; Sun et al. 2025). These systems also support real-time dynamic adjustments to stimuli and task events; automated reward systems deliver rewards precisely within (and across) trials; and scripted (whether fixed, dynamic, or random) inter-trial intervals remove potential timing errors that human experimenters inevitably introduce (Degrande et al. 2022; Lee and Spence 2008; Marquardt et al. 2017; Odland et al. 2021). Their use allows for standardised procedures and reduces experimenter variability in studies where tasks can be translated into screen-based interactions. For certain studies, especially those that require high temporal or spatial precision in task control, automated, bias-resistant methods, such as touchscreen-based setups, offer a practical alternative to reduce operator-dependent variabilities. We acknowledge that full automation is not always necessary or beneficial for every experimental design or research questions, and can still introduce design-related biases, such as those related to stimulus presentation, interface configuration, or reinforcement schedules, which must be carefully controlled, but in the wider context of the reproducibility crisis challenging behavioural science (Johnen et al. 2017; Open Science Collaboration 2015; Spanagel 2022; Wilson et al. 2023), such methods can contribute to enhanced control, consistency, and standardisation.

More specifically, direct and real-time data collected by touchscreen-based systems improves data quality and throughput. Every screen touch is recorded instantly, producing datasets with higher accuracy and lower susceptibility to bias, while reducing delays, expectancy effects, and measurement errors associated with manual procedures (Holman et al. 2015; Hoyt 2000; Michelson et al. 1985; L. E. Miller and Stewart 2011; Rosenthal 1994). Since task progression is determined solely by the subjects’ responses, animals proceed through trials at their own pace without waiting for human input. This design minimises the testing time that would otherwise be lost to operator-related delays, such as manually changing stimuli or re-engaging inattentive subjects. Fully programmed protocols also allow the apparatus to run without continuous human supervision, even inside animals’ home environments, supporting flexible testing while reducing demands on researchers and subjects (Butler and Kennerley 2019; Cabrera-Moreno et al. 2022; Fizet et al. 2017; Huskisson et al. 2021; Nasrini and Hampton 2024).

Integrated timestamping enables synchronisation across behavioural and physiological data streams. We note that precise temporal data are not unique to touchscreen systems. Most automated closed-loop systems as well as many automated computer vision and machine learning approaches can also generate high resolution timestamped data (e.g., Mathis et al. 2018; Pereira et al. 2022; Romero-Ferrero et al. 2019). Within animal behaviour and cognition research, however, many tasks still rely on manual control and coding; in this context, touchscreen paradigms offer a practical and standardised way to automatically log a structured set of task events (e.g., stimulus onset/offset, touches, latencies, inter-response intervals, reward delivery, timeouts) with minimal dependence on real time human annotation. These machine readable events can be used as basis for aligning concurrent video, audio, motion tracking, heart rate, EEG, or other data streams, yielding rich multimodal records that extend beyond aggregate accuracy measures and support modern pattern-mining and modelling approaches (e.g., Gupta 2024; Li et al. 2011; Menaker et al. 2021; Mluba et al. 2024; Saad Saoud et al. 2024; Ye et al. 2023). Collectively, the advantages of touchscreen-based methods respond to current calls for greater rigour and transparency in animal behaviour research (Spanagel 2022; Wilson et al. 2023).

Given these practical advantages of efficiency and flexibility, it is not surprising that touchscreen-based methodologies have been adopted across diverse species, such as gorillas (e.g., Hopper et al. 2021; Truax and Vonk 2023; Vonk 2024; Vonk et al. 2022), chimpanzees (e.g., Gao and Adachi 2024; Huskisson et al. 2020; McEwen et al. 2025; Muramatsu and Matsuzawa 2023; Sato et al. 2023), orangutans (e.g., Gazes et al. 2017; Perdue et al. 2012; Renner et al. 2016; Scheel 2018), capuchin monkeys (e.g., Malassis and Seed 2023; Mendes et al. 2024; Renner et al. 2021), bears (e.g., Bernstein-Kurtycz et al. 2024; Perdue 2016), rats (e.g., Crijns & Op Beeck, 2019), mice (Attalla et al. 2024; Chasse et al. 2023; Palmer et al. 2021), goats (e.g., Gao et al. 2025; Langbein et al. 2023), carrion crows (e.g., O’Hara et al. 2017), pigeons (Blaisdell et al. 2018; Huber et al. 2005; Spetch et al. 1992; Toegel et al. 2021; Wasserman et al. 2013), chickens (e.g., Nasrini and Hampton 2024), and tortoises (e.g., Mueller-Paul et al. 2014). Importantly, their use has not only spanned diverse taxa but has also been reported to be increasingly prevalent in rodents (Dumont et al. 2021) and in nonhuman primates (Egelkamp and Ross 2019) over the past few decades, reflecting both the method’s adaptability and its growing role in comparative cognition (Seitz et al. 2021).

Despite this broad adoption, it remains unclear whether similar growth is occurring in dog research. To address this, the present review had four aims: (1) to investigate whether the upward trajectory of dog behaviour and cognition research continues; (2) to assess whether touchscreen use in dogs is increasing in parallel with both the general dog literature and with touchscreen-based literature using other model species; and (3) to summarise and critically evaluate the main methodological approaches reported in previous touchscreen-based dog studies; and finally, (4) to speculate and discuss the challenges and species-specific considerations related to touchscreen-based research with dogs based on existing evidence.

## Methods

We conducted two literature searches in accordance with the Preferred Reporting Items for Systematic Reviews and Meta-Analyses (PRISMA) guidelines to ensure that our search and article selection process were transparent and replicable (Liberati et al. 2009; Group et al. 2015). The first search, designed to extend the bibliometric trends reported by Aria et al. (2021), targeted general dog behaviour and cognition literature published between 2019 and 2024, thereby updating overall publication trajectories beyond their coverage period. The second search specifically targeted touchscreen-based studies involving dogs across the full available record. The protocol included the following steps: identification (defining search strategies and exclusion criterion), screening and eligibility (selection process), and final inclusion.

### Recent dog cognitive and behavioural studies search

#### Search strategy

We defined the eligibility criteria following the protocol established by Aria and colleagues (2021) to identify dog behaviour and cognition studies, extending the timeframe of the original study beyond 2018 to include all relevant articles published up to (and including) December 2024. The search was conducted on Scopus abstract and citation database (Elsevier; https://www.scopus.com; search date: 5th January 2025; search strategy: (((TITLE-ABS-KEY (((dog OR dogs) AND cogniti) OR (canis AND familiaris AND cogniti))) OR (TITLE-ABS-KEY (((dog OR dogs) AND communicat) OR (canis AND familiaris AND communicat))) OR (TITLE-ABS-KEY (((dog OR dogs) AND behav*) OR (canis AND familiaris AND behav*))))) AND PUBYEAR > 2018 AND PUBYEAR < 2025 AND (LIMIT-TO (LANGUAGE, “English”)) AND (LIMIT-TO (SRCTYPE, “j”)) AND (LIMIT-TO (PUBSTAGE, “final”)) AND (LIMIT-TO (DOCTYPE, “ar”)); retrieved record: 4673 articles).

Aria and colleagues (2021) defined cognitive and behavioural research domains in dogs, and we adopted these domains as our baseline eligibility criteria. To ensure comprehensive coverage, we also interpreted their existing topics and introduced additional topics where necessary, which are marked with an asterisk (*) in Table 1. This step was essential because our search strategy retrieved not only cognitive and behavioural studies but also medical, physiological, and clinical research without direct relevance to cognition or behaviour. Therefore, the inclusion topics outlined below served as a content-based criterion for classifying eligible studies. We included original, peer-reviewed journal articles in English reporting experimental studies on domestic dogs (*Canis familiaris*).

**Table 1:** Topics included in general dog cognitive and behavioural studies (in alphabetical order). * denotes topics newly added or interpreted by us. (adapted from Aria et al. 2021).

Aging (outcomes) as a natural cognitive decline of behavioral
Attention
Behavioral ecology (predation, scavenger behavior, roaming etc.)*
Cranial MRI studies*
Development of new methodology (when using behavioural or cognitive data; such as machine learning algorithms to detect behaviours)*
Domestication (when they deal with cognitive evolution)
Drug treatments or physical therapy (in the case untreated controls are involved; e.g., oxytocin)
Emotions
fMRI studies
Lateralisation
Learning
Memory
Numerical abilities
Perception
Reasoning and problem solving
Spatial cognition
Studies with questionnaires (unless only aimed to study aggression-related behavioural problems, such as biting other dogs)*
Temperament and personality
Theory of mind
Training and working dogs (including assistant and therapy dogs, unless the target is the human benefit)
Welfare and stress in general

#### Exclusion criteria

We excluded review articles and conference proceedings. We excluded studies that focused on other canid species (e.g., African wild dogs, *Lycaon pictus*).

#### Selection process and data collection

**Fig. 1:**
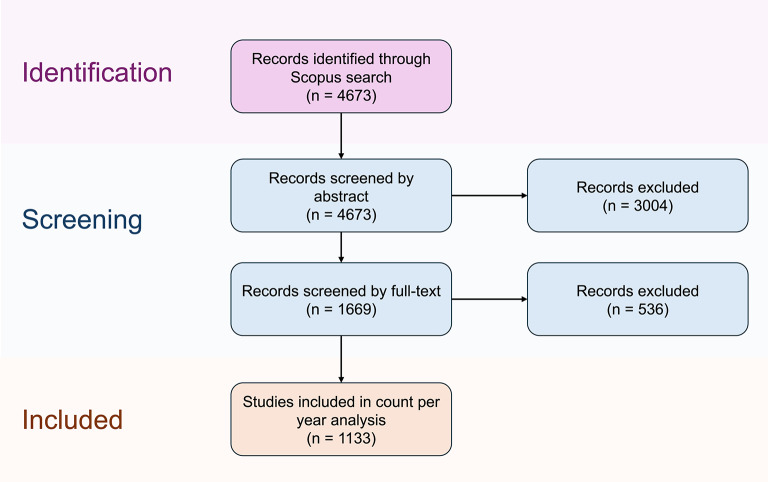
PRISMA flow diagram showing articles on dog behaviour and cognition included and excluded from this review. The diagram summarises the selection procedure for this review following PRISMA guidelines (Group et al. 2015). *Identification*: A total of 4,673 records were identified through Scopus search. *Screening*: 3,540 records were excluded. *Included*: 1,133 studies were included in the count-per-year analysis

The resulting retrieved records were exported in BibTeX format and transferred into Microsoft Excel for further screening to check for inclusion to the dataset. During this stage, articles were divided and reviewed independently by three selectors according to established eligibility criteria for dog behaviour and cognition studies. Any uncertainties or disagreements among selectors were flagged, discussed, and resolved through consensus. Articles meeting the criteria were included in the final dataset (See Supplementary Information and Fig. 1 for a visual depiction of the protocol and the number of articles included/excluded in each stage. Reasons for exclusion for each article can be found in Supplementary Information. Finally, we counted the number of articles in the final dataset per year using basic Excel functions.

## Dog touchscreen-based studies

### Search strategy

We conducted a second search targeting touchscreen-based dog studies. The search was also conducted on Scopus abstract and citation database (Elsevier; https://www.scopus.com; search date: 5th January 2025; search strategy: TITLE-ABS-KEY(((dog OR dogs) AND (“*screen” OR screen OR (touch W/2 screen))).

OR ((canis AND familiaris) AND (“*screen” OR screen OR (touch W/2 screen)))); retrieved record: 8 articles). As in the previous search, we included original, peer-reviewed journal articles in English reporting experimental studies on domestic dogs (*Canis familiaris*).

In contrast to our search for recent dog cognitive and behavioural studies, the Scopus search for touchscreen-based studies using domestic dogs as subject species only returned eight articles. Aiming to capture the breadth of this relatively new and narrowly focused topic, we conducted backward (by checking the reference list of retrieved studies) and forward citation chasing (by checking newer studies that have cited the retrieved articles), also referred to as snowball sampling, to identify additional touchscreen studies (Badampudi et al. 2015; Booth 2016; Higgins and Thomas 2019).

### Exclusion criteria

We excluded review articles and conference proceedings. We excluded studies that focused on other canid species (e.g., African wild dogs, *Lycaon pictus*). We also excluded any publications that used alternative controllers (e.g., joysticks, buttons, handles) rather than direct screen touches.

### Selection process

**Fig. 2:**
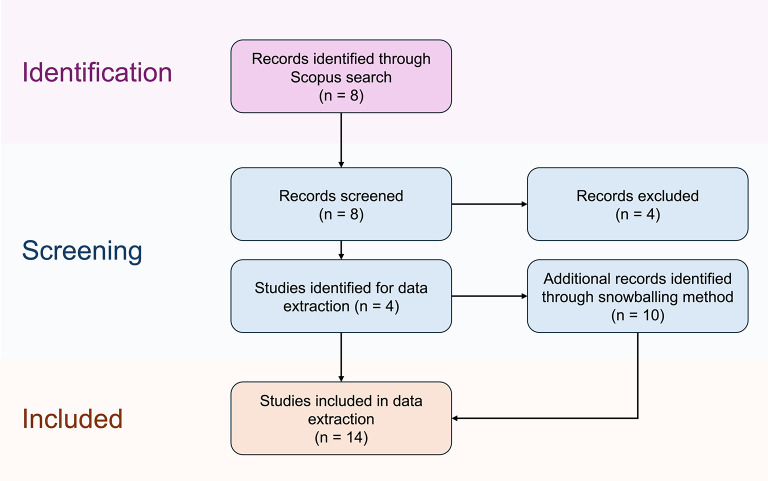
PRISMA flow diagram showing articles using touchscreen-based methods with dogs included and excluded from this review. The diagram summarises the selection procedure for this review following PRISMA guidelines (Group et al. 2015). *Identification*: A total of eight records were retrieved from database searches. *Screening*: four were excluded, and ten additional records were identified through snowballing. *Included*: fourteen studies met the inclusion criteria and were included in the final data extraction

The resulting retrieved records were exported in BibTeX format and transferred into Microsoft Excel for further screening to check for inclusion to the dataset. Articles meeting the criteria were included in the final dataset. The protocol used for this search and articles included in each stage were illustrated in Fig. 2.

### Data collection

We counted the number of articles in the final dataset per year using basic Excel functions. Methodological details and results from the final dataset were manually extracted: authors; year of publication; type of touchscreen; recruited subject number; included subject number; dropped-out subject number (i.e., dogs lost for reasons outside experimental control, such as owner unavailability, dog illness, or death); excluded subject number (i.e., dogs that failed to learn the task, showed persistently low motivation, or disengaged during training); type of stimulus; training schedule; training frequency; training duration; pre-training required (session number); training required (session number); subject population (pet or lab dog); and previous touchscreen experience. We also calculated meta-results: inclusion rate (included subject number/recruited subject number), and pre-training and training time (sessions).

## Results and discussion

**Fig. 3:**
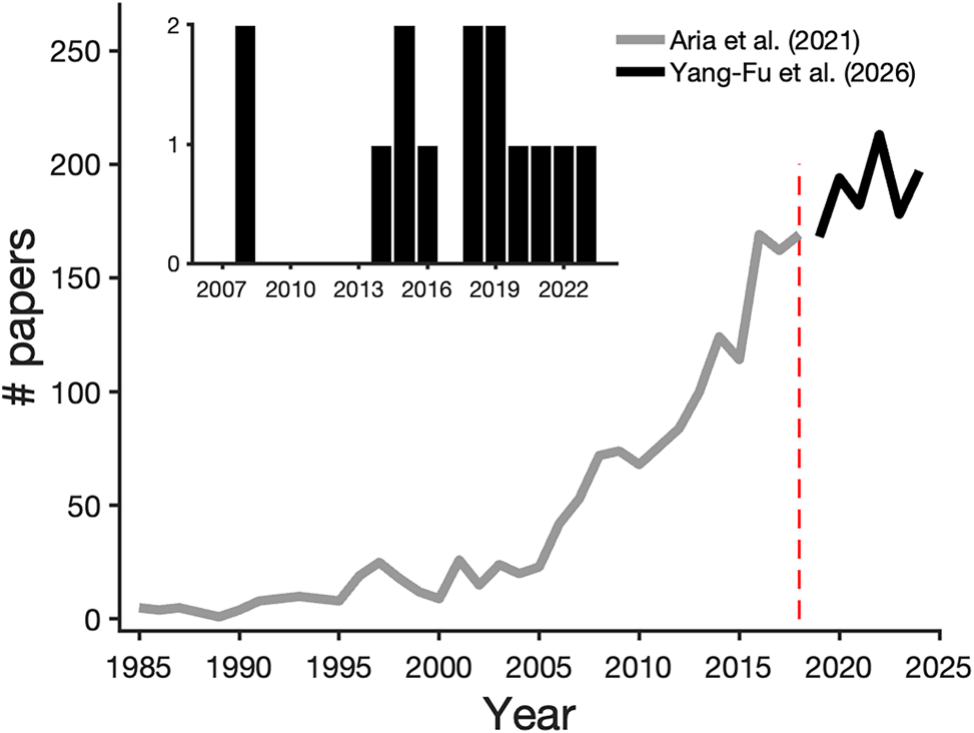
Number of dog cognitive and behavioural studies show a sustained and increasing trend. Grey and black lines depict the data reported in Aria et al. (2021; data covering 1985–2018) and from our search (covering 2019–2024), respectively. 1133 articles were included in the final dataset for recent dog cognitive and behavioural studies. The complete list of included articles can be found in Supplementary Information**.** Inset shows dog studies that have used touchscreen-based methods. Created using Matlab 2024b

The comparison between the number of publications on dog behaviour and cognition and touchscreen-based dog studies highlights the disparity between the two (Fig. 3). While the general field of dog behaviour and cognition has shown a continuous growth, with hundreds of articles being published every year, touchscreen-based dog studies remain very limited in number — there have only been fourteen touchscreen-based articles since 2007 (Fig. 3, inset and Table 2). Importantly, our search strategy for touchscreen dog studies was deliberately more inclusive than the general literature count, extending beyond database search to citation chasing, because the initial database search returned very few records. This asymmetry in method supports our observation that even after applying broader and more comprehensive retrieval procedures designed to increase coverage, the touchscreen dog literature remains limited relative to the rapidly expanding general dog behaviour and cognition field.

These results show that the number of publications in dog behaviour and cognition continues to rise, reflecting the growing interest in using dogs as a model for these lines of research, consistent with what has been reported previously (Aria et al. 2021), supporting our first hypothesis. However, contrary to our expectations, our search revealed only a very limited number of studies that used touchscreen-based methodologies with dogs. This finding indicates that, despite the increasing number of dog studies using other methods, touchscreen-based approaches remain underutilised.

**Table 2:**
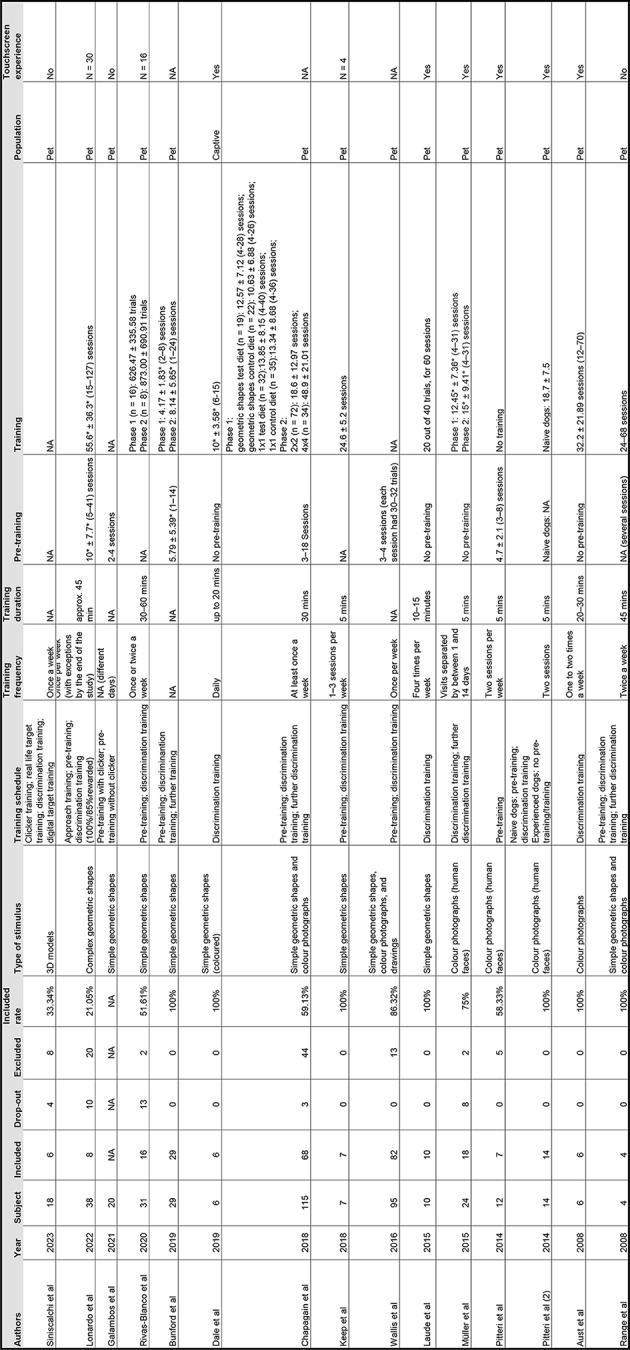
Methodological and training details for dog touchscreen-based studies

* denotes data calculated by us based on original raw data.* Pre-training* generally refers to familiarising dogs with the experimental apparatus or basic task demands (e.g., touching a given stimuli or touching the screen). *Training* typically involves teaching dogs to distinguish between two stimuli (e.g., shapes, faces, or quantities), with correct choices reinforced. Terminology varies between studies. We contacted corresponding authors for additional data not reported in the original manuscripts. Cells marked as N/A indicate information that was either no longer accessible or for which no reply was received by the time of submission

In the sections that follow, we discuss a few insights from the fourteen touchscreen-based dog studies included in our review. We then speculate on potential barriers to the wider adoption of touchscreen-based methods in dog research and consider their methodological limitations. Finally, we propose strategies to mitigate these challenges and offer recommendations for future studies intending to incorporate touchscreen-based approaches.

### Existing touchscreen-based dog studies

Among the fourteen publications of touchscreen-based studies using domestic dogs, several overarching principles emerged. To start, multi-stage training is universal. The majority of studies described incremental training programmes in which dogs first learned to orient toward the apparatus, then to touch a single stimulus, and only subsequently asked to discriminate among alternative options. Training schedules ranged from a few sessions (Aust et al. 2008; Pitteri et al. 2014), to multiple visits per week (e.g., Laude et al. 2016), to extended training regimes exceeding more than one hundred sessions (Lonardo et al. 2022), suggesting that reliable screen interaction is rarely achieved in a short time frame (Table 2). The fact that most studies required lengthy multi-stage training highlights both the critical role of training for experimental success and the substantial investment of time and personnel it demands. These demands become even more pronounced when the subjects are pet dogs, who live in private homes with owners, and it is simply impractical to test them with the same frequency as lab animals, which we will further discuss below.

**Fig. 4:**
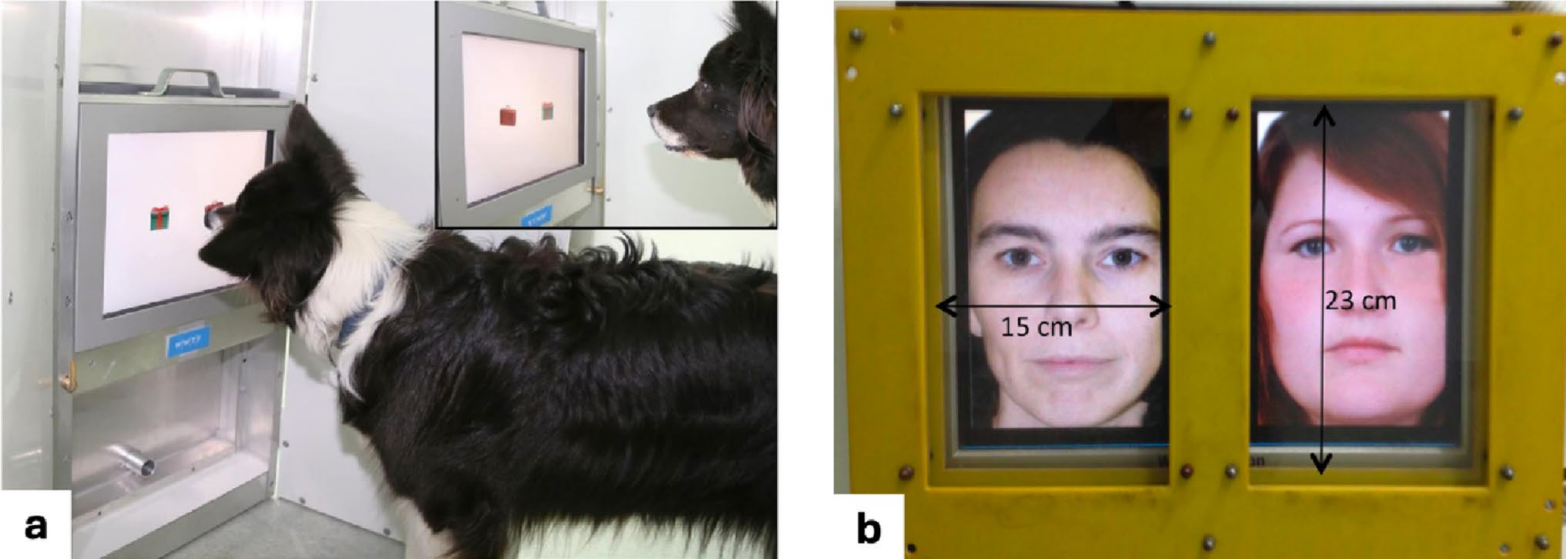
Comparison of open-design and constrained-design touchscreen apparatuses used in dog studies. (**a**) Open-design set-up in which the full touchscreen is accessible to the dogs (adapted from Wallis et al. 2016); (**b**) Constrained-set-up in which the touchscreen is placed behind physical barriers with only defined response windows presented to the dogs (adapted from Pitteri et al. 2014). Images used (cropped for layout) under the Creative Commons Attribution license (CC BY 4.0; https://creativecommons.org/licenses/by/4.0/)

Apparatuses’ set-ups fall into two general categories: open-design and constrained-design (Fig. 4). In open-design apparatuses, the entire touchscreen is accessible to the dog (e.g. Siniscalchi et al. 2023; Wallis et al. 2016) allowing flexible placement and real-time relocation of multiple stimuli, which facilitates paradigms that require more than two response options or spatially defined metrics (Fig. 4a). In constrained design apparatuses the touchscreen is placed behind physical barriers such as metal frames or wooden boards (Fig. 4b), exposing a pair of response windows (Müller et al. 2015; Pitteri et al. 2014; Rivas-Blanco et al. 2020). By limiting access to task-relevant areas, this configuration inherently prevents interactions with irrelevant screen areas, thereby simplifying the task into binary response structure and reducing accidental irrelevant touches. Therefore, the design of the apparatus should be selected based on the research question under inquiry. Researchers interested in multi-choice arrangements, dynamically shifting stimuli (e.g., moving patterns), or flexible spatial analysis should benefit from an open design. In contrast, projects that only require binary discriminations or choices could employ the constrained design.

The stimuli used across studies varied widely. Roughly half of the studies surveyed used geometric shapes, while the remaining employed colour photographs, human faces, or mixed icon sets (Table 2). While this diversity highlights the flexibility of touchscreen-based platforms, using different stimulus types could impact dog engagement, learning rates, and ultimately differently affect the underlying cognitive processes (Hirskyj-Douglas et al. 2017).

Studies also varied in terms of the level of human involvement. In some, the handler stood beside the dogs, holding them back during the inter-trial interval and releasing them when choice became available (Müller et al. 2015; see later for a discussion of potential reasons for requiring a human experimenter). While this practice can prevent undesired screen touches, it reintroduces potential experimenter cues. Even when the handler cannot see the screen or the stimuli, their physical interaction with the dog, such as the timing, firmness, or subtle hesitation in restraint and release, may inadvertently signal information that influences the animal’s choice. For example, if a dog anticipates that the experimenter’s brief hold or delayed release indicates an incorrect approach, it might adjust its behaviour accordingly, leading to choices guided by perceived social feedback rather than independent discrimination. Such unintentional cues compromise the objectivity of the data, invalidate measures like response latency, and may shift the dog’s attentional focus toward the human rather than the task itself. These factors complicate cross-site replication and limit the validity of results, as performance in such setups may no longer accurately reflect autonomous decision-making. In other studies, fully autonomous apparatus operate with no experimenter present (Siniscalchi et al. 2023), thereby minimising social influence, but requiring more robust hardware and potentially more training with the dogs to shape and maintain adequate behaviours.

Participants populations were mostly pet dogs, with one study that tested pack dogs kept in enclosures (Dale et al. 2019). Notably, more than half of the articles reported that at least some of their subjects had prior experience with touchscreen experiments. Yet only one had specific information about it (Aust et al. 2008 tested dogs that also participated in Range et al., 2007). The observation that several articles mentioned previous touchscreen experiences without citing corresponding published studies prompted us to speculate that some touchscreen-based experiments may have been conducted but never reported. This potential ‘file-drawer’ effect will be discussed further in following section.

Training schedules varied considerably across the studies surveyed. Some laboratories conducted intensive daily sessions (e.g., Dale et al. 2019; Table 2), while others scheduled training sessions more flexibly (e.g., Keep et al. 2018; Table 2). The frequency and total duration of training have been suggested to influence the rate at which animals acquire and retain learned material (Demant et al. 2011; Heinrich et al. 2020; Meyer and Ladewig 2008; Smith et al. 2025). For instance, studies have reported that for acquisition and memory in conventional obedience work, training twice per week would be optimal (Demant et al. 2011); and two short sessions separated by a five-minute break can optimise performance in touchscreen discrimination tasks (Range et al. 2008). As these recommendations are based on specific study design and limited datasets, they are suggestive rather than prescriptive. Where consistency across testing sessions is impractical, detailed reporting of inter-session intervals will enable subsequent meta-analyses to investigate how schedule structure influences results and how it interacts with other control factors (e.g., age, breed and motivational state).

Interestingly, during our search, we came across a subset of studies that used semi-automated approaches. For instance, Byosiere and colleagues tested visual perception in dogs by displaying stimuli on a monitor and automatically recording nose-touches, but task control such as sample presentations and trial contingencies remained under human control (Byosiere et al. 2017, 2018a, b, 2019a). These setups reduce some experimenter influence by standardising response logging, yet the remaining manual procedures can still introduce subtle biases and imprecisions. Nonetheless, semi-automation may be a pragmatic solution for laboratories lacking the resources or expertise to implement full touchscreen automation. Despite the constant reduction in the price of hardware components, the initial investment might still be unaffordable for many facilities, and the design choices evident in these studies offer valuable insight into incremental paths toward reduced experimenter involvement.

In addition to the journal articles included in our dataset, we note two relevant conference proceedings. Zeagler and colleagues (2014) reported that dogs’ touchscreen inputs often resemble swipes or dragging rather than discrete taps, and that infrared design can be reliable to dogs’ wet noses contacts, whereas some common alternatives (e.g., capacitive approaches) can be problematic in dog use. Byrne and colleagues (2018) demonstrated the practical feasibility of in-home touchscreen deployment by training assistance dogs to execute an emergency action on a touchscreen. They showed that dogs can learn and perform a multi-step icon sequence with high reliability in a naturalistic home setting. Taken together, these studies illustrate an incremental pathway toward reliable, dog-centred systems that can be used to study various questions, thereby informing the recommendations we outline below.

### Barriers and prospective routes to wider adoption of touchscreen-based methods in dog research

The studies reviewed here provide a basis for identifying why touchscreen-based methodologies remain underutilised in dogs and for extracting actionable design and implementation guidance. Below, we organise the discussion into key barriers and potential solutions, drawing not only on the dog touchscreen literature but also on relevant work from broader dog cognition, animal–computer interaction/human-computer interaction, and touchscreen-based research in other model species.

### Technical difficulties

Implementing a dog-proof touchscreen apparatus requires a combination of specialised know-how across multiple domains, including knowledge of or experience with working with the target animal species, proficiency in programming languages for task control, and some degree of engineering and/or technical skills for hardware development and troubleshooting (Ajuwon et al. 2024a, b; Kravitz and Laubach 2024). From the interface side, researchers must design dog-appropriate systems that respond effectively, to facilitate learning, which can be difficult as dogs interact with the screen with their noses and/or paws. If the apparatus fails to respond when needed, it makes task contingencies unreliable, which may cause frustration, disengagement, or slow learning. Hardware choices also involve trade-offs. For example, projected capacitive screens are susceptible to moisture, producing false or inaccurate touches from wet noses or when in contact with saliva. Protective overlays or water-resistant designs can reduce these problems, but they add cost and weight, which is relevant for laboratories that value portability (e.g., mobile or home-deployable setups).

### Solutions for technical difficulties

Open-source hardware and software solutions provide affordable options for laboratories wishing to incorporate touchscreen-based tasks without reinventing apparatus design from scratch. Numerous studies have demonstrated that self-built computer-controlled apparatuses, created from combinations of custom-made (e.g., laser cut and/or 3D printed parts) and off-the-shelf components, can be successfully adapted to a variety of species (Ajuwon et al. 2024a, b; Arce and Stevens 2022; Buscher et al. 2020; Butler and Kennerley 2019; Devarakonda et al. 2015; O’Leary et al. 2018; Pineño 2013). Furthermore, the ongoing reduction in the cost of digital devices and hardware fabrication (Pearce and Qian 2022; Rayna and Striukova 2021) means that the costs associated with building touchscreen-based setups are gradually decreasing (Oxley et al. 2022). Repositories such as GitHub (https://github.com*)* and OpenBehavior (https://edspace.american.edu/openbehavior/*)* also reduce the technical learning demands for scientists with limited engineering or programming experience by providing task-control scripts (e.g., stimulus presentation and response logging), circuit diagrams, and design schematics that are re-usable across projects.

Dog researchers can draw insights and inspiration from the human-computer interaction literature. Human-computer interaction has a long history of research on how users perceive, remember, and respond to screen-based stimuli (e.g., Hutchins et al. 1985; Norman 1999; Shneiderman and Plaisant 2005). These principles stress the importance of feedback, visibility, and intuitive mapping between action and outcome, considerations that are just as relevant when dogs are the ‘users.’ For example, studies of pointing and movement (Bi et al. 2013; Fitts 1954; Parhi et al. 2006) demonstrate that target size and spacing affect the accuracy and speed of selections, suggesting that using larger, well-spaced, and visually distinct stimuli is likely to facilitate dogs’ interaction with stimuli on the screen. Similarly, research on gesture recognition and error prevention has proposed strategies that emphasis accessibility of technology and how it should and could be designed to make interactions easier for the users (e.g., Khan and Khusro 2019; Wigdor and Wixon 2011; Wobbrock et al. 2011; also see such investigation in dogs: Hirskyj-Douglas et al. 2017; Zeagler et al. 2016 [note: Zeagler et al. examined touchscreens, while Hirskyj-Douglas et al. investigated interactions towards screens in general]). Applying these insights from human-computer interaction studies to dog-specific contexts could potentially improve reliability, reduce frustration, and assist learning.

We also suggest establishing collaborative frameworks across laboratories for touchscreen-based studies in dogs to standardise and verify touchscreen design decision. Multi-lab initiatives have demonstrated that shared protocols, open materials, and transparent reporting can accelerate method refinement and improve reproducibility (Alessandroni et al. 2024; Altschul et al. 2021; Byers-Heinlein et al. 2020; Coles et al. 2022; De Moor et al. 2025; International Brain Laboratory & International Brain Laboratory 2017; Lambert et al. 2022; Lucca et al. 2025; ManyDogs et al. 2021; ManyGoats 2024; ManyPrimates et al. 2020; Rance 2024). By investing in similar multi-lab collaborative models specifically tailored to dog touchscreen-based research, the community could systematically refine experimental procedures, validate protocols within and across dog populations, and develop benchmarks for training, testing, data sharing and analyses. Such collaborative efforts could foster a culture of open data and produce shareable ‘survival guides’ that include step-by-step software and hardware build instructions and troubleshooting manuals, and can substantially reduce study timeframes, while making a more substantial contribution to the cumulative scientific progress within dog cognition research.

### Practical constraints of costs, labour, and publication bias

Technical demands described above translate into significant financial and personnel costs. As successfully adopting touchscreen-based methods require interdisciplinary skills, it might require extensive training, effective collaboration among researchers from diverse fields, or attracting individuals who already possess this multifaceted skill set; potentially challenging given the increasing trend of talent migration from academia to industry roles, driven by better compensation and life quality (Shao et al. 2024; Torrisi and Pernagallo 2020; Woolston 2020).

A touchscreen-based apparatus requires initial investment. Building such apparatus normally involves (at minimum) a touch-capable monitor, an automated feeder, and a computer. Based on commercial material prices and published studies, the minimal costs for a complete set-up would be around 800 Euros, and depending on the system’s design, brand, and customization, the cost can exceed 10,000 Euros (e.g., Horner et al. 2013; Steurer et al. 2012). While this investment can be amortised across many studies (reducing cost per session over time), it is substantially higher than that of many manual experiments, which may require little more than basic materials such as pen, paper, or simple objects.

Extensive training, sometimes more than a hundred sessions (e.g., Lonardo et al. 2022), requires substantial labour not only from researchers but also time investment and availability from owners accompanying their pet dogs to the lab. Moreover, current dog touchscreen protocols still require supervision to manage the session and monitor animal welfare, limiting the degree to which training can be fully automated.

Finally, these costs interact with publication bias, as slow training trajectories, unsuccessful pilots, or failures do not reach publication, generating a file-drawer effect (Greenwald 1975; Rosenthal 1994; Simonsohn et al. 2014). Consequently, the literature likely over-represents successful implementations and under-represents the true distribution of training timelines, feasibility constraints, and effect sizes, potentially discouraging adoption through unrealistic expectations or repeated reinvention of unproductive designs.

### Practical solutions

Survival guides discussed above would also help to reduce the financial and personnel demands. Detailed shopping lists (i.e., build of materials, BOM), assembly protocols and checklists, would allow laboratories with limited engineering capacity to assemble and deploy touchscreen-based methodologies and at reduced price points.

Standardised touchscreen-based training schedules and stimuli set for dogs can also streamline training, reducing the time spent designing and piloting protocols. Crucially, a shared repository should invite uploads of all outcomes (including null or negative results), enabling cumulative learning about what fails, under what conditions, and at what cost. Such a shared repository directly addresses the file-drawer problem and reduces duplicated effort across laboratories.

When such open-source designs are made portable or autonomous, testing can shift closer to dogs rather than requiring dogs to travel to labs, reducing owner burden and expand sampling. Home-based apparatuses would enable owner-assisted sessions, expanding geographic reach (although this may require more experimental units), and their practical feasibility has been demonstrated in an in-home touchscreen deployment with assistance dogs (Byrne et al. 2018). Even though participant recruitment remains voluntary, it could support a new form of citizen science, where owners engage in cognitive testing with their dogs, contributing to larger datasets while enriching their animals’ daily routines. Conversely, installing apparatuses in kennels or facilities with captive dogs would create continuous data streams from larger cohorts under controlled conditions (Griggs et al. 2021; Harrison et al. 2023; Martin et al. 2022; Schmitt 2018). In both scenarios the logistical burden shifts away from lab personnel, shortens start-up time and diversifies sampling beyond the typical ‘owners who can drive to campus’ demographic, mitigating prevalent recruitment biases (e.g., Ganguli et al. 2015; Oswald et al. 2013; Rosnow and Rosenthal 1976).

### *Umwelt* concerns

Beyond technical and practical barriers, touchscreen-based research with dogs must also consider the alignment between the experimental interface and dogs’ perceptual systems and motor capabilities -- their *Umwelt* (Uexküll 2001; Uexküll and Kriszat 2013). Here we use the concept of *Umwelt* to refer not only to an animal’s sensory abilities, but also to the bodily and motor capacities through which it interacts with its environment, as well as the ecological and social contexts that shape their daily life. For dogs, this includes their reliance on other sensory cues (e.g., olfaction) in addition to vision, the physical ergonomics associated with using a nose on a flat, vertical, screen, and their social role as human companions that constrains how and when they can be tested in laboratory settings. Cognitive characteristics, such as dogs’ varying levels of inhibitory control, also form part of this species-specific *Umwelt* and influence how easily individuals can adapt to touchscreen-based tasks.

Screen-based paradigms, including touchscreens, share general display constraints due to dogs’ lower acuity (Byosiere et al. 2018a, b; Lind et al. 2017; P. E. Miller and Murphy 1995), dichromatic vision (Siniscalchi et al. 2017), and a higher flicker-fusion threshold (Coile et al. 1989; Hirskyj-Douglas et al. 2017; Sheldon et al. 2024). Empirical data on canine luminance and colour sensitivity also remain limited (but see (Byosiere et al. 2018a, b, 2019b; P. E. Miller and Murphy 1995; Neitz et al. 1989). These considerations are not unique to touchscreens, but they limit what any monitor-presented stimulus can effectively test.

Motorically, dogs are not naturally inclined to interact with vertical, two-dimensional surfaces using their noses. Many behaviourally relevant object properties are absent or only symbolically represented on a display: a nose-touch on a screen lacks the olfactory, haptic, and proprioceptive cues that normally guide exploratory or social behaviours (e.g., texture, temperature, compliance, and odour). Performance on 2D representations can vary widely across individuals even when discrimination is possible (e.g., Autier-Dérian et al. 2013; Mongillo et al. 2021; Pongrácz et al. 2003; Range et al. 2008). Additionally, although no studies have concluded sufficiently large or diverse samples to robustly test for breed differences in touchscreen interactions, it is likely that these perceptual and behavioural factors vary across breeds due to physiological differences. Ergonomics also matter, as the spatial offset between eyes and nose tip means that the attended location may not match the contact point of the nose on screen, and this offset varies with morphology (e.g., short-headed compared to medium- or long-headed breeds (Canori et al. 2025; Ichikawa et al. 2024), with some individuals or breeds might need to learn awkward or uncomfortable positions during repeated trials. Additionally, it has been reported that some dogs interact with the screen by sliding or swiping nose from one target to another rather than making clean taps (Zeagler et al. 2016). This observation questions whether the default tap-only interaction, derived from human interface design, is the most intuitive or efficient input method for dogs.

Furthermore, pet dog testing contexts constrain exposure frequency. Unlike laboratory-maintained models that can complete hundreds of trials per day, 5 to 7 days a week (e.g., Alsiö et al. 2019; Fagot and Paleressompoulle 2009; Horner et al. 2013),, pet dogs often attend only a few sessions per week, limiting training throughput. This interacts with the lengthy training often required for touchscreen use mentioned before, two predictable outcomes follow. First, researchers might limit themselves to research questions that can be answered quickly or with minimal training, narrowing the scope of the studies that are deemed feasible to be conducted with dogs. Second, studies that turn out to be slower or less productive within the expected timeframe might stall before publication, contributing to the publication bias described earlier.

Touchscreen-based paradigms may introduce an additional layer of selection of subjects. Because participation requires criterion-based training, individuals who fail to acquire the task (or who disengage during training) are often excluded from subsequent testing. This exclusion creates the possibility that the final tested sample is systematically biased toward individuals with particular physiological, behavioural or cognitive characteristics (e.g., higher trainability, persistence, lower neophobia, stronger food motivation), thereby limiting generalisability to the broader population (e.g., Barnard et al. 2018; Lazzaroni et al. 2019). This issue aligns conceptually with the gifted word learner subpopulation in word-learning research, where only a small subset of dogs shows exceptional acquisition and retention of object labels (Dror et al. 2024, 2026; Fugazza et al. 2021). In the dog touchscreen studies included in our review, we did not find systematic examinations of learner-non-learner differences or analyses of how training shapes the resulting sample, likely reflecting the small sample sizes that characterise this literature.

Additionally, laboratory studies typically benefit from consistent rearing history of animals and controlled schedules for feeding, activity, and testing; such standardisation, however, is not feasible with pet dogs living in private households. Researchers have no control over their day-to-day routines, recent exercise, or food intake, which can vary widely across dogs. Touchscreen-based studies rely primarily on food as the reinforcer, and researchers can only request that owners withhold food for a certain period before a testing session; compliance and exact timing inevitably vary. This variability reduces experimental control over motivation, inflates within- and between- subject noise, and can lengthen training.

Another potential problem is that the inhibitory control of dogs is context-dependent, varying depending on the task, social context, and testing paradigm (Bray et al. 2014; Brucks et al. 2017a, b; Fagnani et al. 2016a; Marshall-Pescini et al. 2015; Vernouillet et al. 2018). For example, in a large comparative across 36 species, dogs were ranked in the mid-range overall, but their performance differed markedly between inhibitory control paradigms, with higher impulsivity scores in cylinder tasks compared to in A-not-B tasks (MacLean et al. 2014). This variation supports that inhibitory control in dogs is not a stable trait but can depend strongly on the specific task structure. Social contexts matter too, as dogs performed better in a social version of an inhibition task with humans present than in a non-social version (Fagnani et al. 2016b; but also see Bray et al. 2015). Touchscreen-based studies, however, make the opposite demand: once the training is complete, the dog must work without human help, choosing whether and when to engage or withhold a nose-touch in front of a screen. It is likely that dogs have difficulties withholding impulsive nose-touches once they know how to touch the screen, human handlers therefore end up restraining the dog or physically restricting the screen to prevent unwanted touches during the inter-trial intervals or before stimulus presentation. Currently it remains unclear whether the apparent ‘impulsivity tax’ associated with touchscreen use reflects a genuine limitation in dogs’ inhibitory control, or whether it arises from task design factors, such as the removal of human social cues, that normally support inhibition during manual testing.

A related and critical challenge is ensuring the dogs’ attention is directed at the stimulus itself. It can be difficult to confirm that the dog is not just interacting with the apparatus but is actively processing the visual or auditory cues presented. This lack of attention can prolong learning, as a subject might touch the screen impulsively without looking at the stimulus, yet still receive reinforcement for a technically correct, yet random, response.

### *Umwelt* considerations

Aligning task design with dogs’ perceptual capacities begins with using stimuli that dogs can reliably see and discriminate. Because dogs perceive colour through a dichromatic (blue-yellow) system, stimuli differing strongly along this spectrum (e.g., deep blue vs. vibrant yellow, or black vs. yellow) are likely to be more easily distinguishable than contrasts that rely primarily on human red-green distinctions (P. E. Miller and Murphy 1995; Pongrácz et al. 2017; Siniscalchi et al. 2017). Discriminability also depends on luminance, contrast and saturation, so colour manipulations should be piloted with different visual parameters controlled where possible (Siniscalchi et al. 2017; see also Byosiere et al. 2019a). Notably, dogs can sometimes succeed on red-green discriminations under specific stimulus configurations, particularly when luminance is carefully controlled, suggesting that performance depends on precise stimulus properties and task design (Byosiere et al. 2019b; P. E. Miller and Murphy 1995; Neitz et al. 1989; Pongrácz et al. 2017; Siniscalchi et al. 2017).

For stimuli with motion, display refresh rates should be chosen with dogs’ temporal resolution in mind (Abdai 2025; Lõoke et al. 2020; Lorenzi and Vallortigara 2021; Scholl and Tremoulet 2000; Schultz and Frith 2022; but also see Kanizsár et al. 2017). Screens that appear stable to humans may shimmer to dogs if refresh rates are too low, potentially degrading stimulus fidelity (Coile et al. 1989). Motion may increase stimulus salience against static backgrounds (e.g., Waldin et al. 2017), although this remains to be empirically tested in dogs. Before committing to a final stimulus set, pilot studies that vary size, contrast, colour composition, and motion should ensure that dogs can, in fact, detect the different experimental contingencies.

To provide the screen with more sensory feedback that typically guides a dog’s exploratory behaviour, visual events are often paired with secondary feedbacks such as brief auditory tones or inter-trial signals in touchscreen-based dog studies (e.g., Laude et al. 2016; Rivas-Blanco et al. 2020). Different forms of secondary reinforcement have been studied in dog training, including the use of auditory cues such as clickers, verbal signals, and different reinforcement schedules aimed at bridging the temporal gap between a correct response and the delivery of food (e.g., Cimarelli et al. 2021; Feng et al. 2016; N. J. Hall et al. 2013; Lazarowski et al. 2025). Empirical findings have been mixed regarding the use of clickers, with some studies found little or no advantage of clicker training compared to conventional food-only reinforcement (e.g., Burton 2020; Gilchrist et al. 2021), whereas Lazarowski et al. (2025) reported clear benefits of clicker in detection dog training. They attributed this difference to methodological and contextual factors, specifically the temporal gap between behaviour and reward delivery, task complexity, and trainer expertise. Markers appear most advantageous in conditions requiring precise timing, when the task is difficult, or when immediate primary reinforcement might be delayed, supporting its function as an effective secondary reinforcer in touchscreen studies. It is also possible to connect the touchscreen with external devices such as buttons or capacitive paw pads (e.g., Kenawell et al. 2025), aligning the visual presentation of stimuli with the motor act associated with natural exploratory movements. Related work has likewise developed touchscreen for medical alert and wearable interfaces for working dogs, emphasising designs that remain usable under constraints and that leverage behaviours dogs can perform comfortably and reliably (Jackson et al. 2018).

Practical interface should include ergonomic accommodation and design for nose-based input, such as generous target sizes, wider spacing, and tolerance for sliding contacts, rather than assuming discrete tap events (Zeagler et al. 2016). Where feasible, software should treat contact trajectories as meaningful data (not merely noise), both for error analysis and for understanding dogs’ motor capabilities.

To address potential sampling bias, studies should compare baseline characteristics of dogs that reached final testing stage versus excluded dogs. This strategy aligns with broader calls to improve external validity in behavioural science (e.g., STRANGE considerations; Webster and Rutz 2020), and would allow the field to quantify whether touchscreen paradigms favour particular dog profiles.

Regarding inhibitory control, studies can incorporate mechanisms such as limited hold responding, variable inter-trial-intervals, and timeouts for impulsive touches (responses before stimuli presentation), drawing conceptually from rodent touchscreen protocols (e.g., Beraldo et al. 2019; Kim et al. 2015). These measures can both reduce impulsive touches and generate quantifiable metrics of inhibitory control or attentional failures.

A direct way to test the social support hypothesis is to implement paired conditions in which dogs perform the same touchscreen task with and without human presence. This comparison would clarify whether inhibitory control problems are intrinsic to touchscreen use or are partially a byproduct of removing social cues that facilitate response inhibition.

To ensure attention to the stimulus, and to handle cases where the dog makes incidental touches or does not respond at all, touchscreen paradigms are typically implemented as closed-loop systems, in which the animal’s ongoing behaviour (e.g., response initiation, touch timing, location, and trajectory) determines whether a response is accepted and whether the trial advances. Actionable design suggestions to further improve the touchscreen-based task design have been proposed in other species’ literature. In classic touchscreen research, selection can be defined at initial contact, at the first contact with a target, or on release, with lift-off selection allowing continuous feedback and correction before committing a response (Potter et al. 1988). These distinctions are especially relevant for dogs, where sliding on screen is common (Zeagler et al. 2014). A study with parrots also reported frequent multi-contact and recommended intervention by using larger targets and filtering excessive touches with software to improve selection reliability (Kleinberger et al. 2024). In macaques, protocols require responses on the restricted areas where stimuli are presented and penalize touches on blank areas (e.g., Loyant et al. 2022). Similarly, chickens are required to peck the same stimulus multiple times to confirm their selection and distinguish deliberate choices from accidental or clumsy touches (e.g., Nasrini and Hampton 2024). We acknowledge that not all design choices can be directly transferrable to dog. Precision requirements, such as responding to a narrowly defined touchscreen area, may need adjustment to accommodate a dog’s nose touches.

In conclusion, touchscreen research with dogs is rare not because it lacks value, but because it faces technical, practical, and species-specific challenges. Potential solutions are proposed in this review. By adopting open-source designs, sharing detailed building and training guides, and developing common reporting standards, researchers can make touchscreen studies more accessible and comparable across labs. Multi-lab collaborations and home-based setups could further expand participation and data diversity. With collective effort and dog-centred design, touchscreen methods can become a standard, reliable tool for studying canine behaviour and cognition, improving reproducibility and accelerating discovery.

## Supplementary Information

Below is the link to the electronic supplementary material.

Supplementary Material 1

## Data Availability

All data is available as supplementary material.

## Declarations

### AI statement

We have not used AI-assisted technologies in creating this article.

## References

[CR1] Abdai J (2025) Perception of animate motion in dogs. Front Psychol 15:1522489. 10.3389/fpsyg.2024.1522489 39830849 10.3389/fpsyg.2024.1522489 PMC11739167

[CR2] Ajuwon V, Cruz BF, Carriço P, Kacelnik A, Monteiro T (2023) GoFish: a low-cost, open-source platform for closed-loop behavioural experiments on fish. Behav Res Methods. 10.3758/s13428-022-02049-2 36622558 10.3758/s13428-022-02049-2 PMC10794453

[CR3] Ajuwon V, Cruz BF, Carriço P, Kacelnik A, Monteiro T (2024a) GoFish: a low-cost, open-source platform for closed-loop behavioural experiments on fish. Behav Res Methods 56(1):318–329. 10.3758/s13428-022-02049-2 36622558 10.3758/s13428-022-02049-2 PMC10794453

[CR4] Ajuwon V, Cruz B, Monteiro T (2024b) GoFish: a foray into open-source, aquatic behavioral automation. J Fish Biol. 10.1111/jfb.15937 39313915 10.1111/jfb.15937 PMC12536047

[CR6] Alessandroni N, Altschul D, Baumgartner HA, Bazhydai M, Brosnan SF, Byers-Heinlein K, Call J, Chittka L, Elsherif M, Espinosa J, Freeman MS, Gjoneska B, Güntürkün O, Huber L, Krasheninnikova A, Mazza V, Miller R, Moreau D, Nawroth C, Prétôt L (2025) Challenges and promises of big team comparative cognition. Nat Hum Behav 9(2):240–242. 10.1038/s41562-024-02081-6 39695249 10.1038/s41562-024-02081-6

[CR5] Alessandroni N, Altschul D, Bazhydai M, Byers-Heinlein K, Elsherif M, Gjoneska B, Huber L, Mazza V, Miller R, Nawroth C, Pronizius E, Qadri MAJ, Šlipogor V, Soderstrom M, Stevens JR, Visser I, Williams M, Zettersten M, Prétôt L (2024) Comparative cognition needs big team science: how large-scale collaborations will unlock the future of the field. Comp Cognit Behav Rev 19:67–72

[CR7] Alsiö J, Phillips BU, Sala-Bayo J, Nilsson SRO, Calafat-Pla TC, Rizwand A, Plumbridge JM, López-Cruz L, Dalley JW, Cardinal RN, Mar AC, Robbins TW (2019) Dopamine D2-like receptor stimulation blocks negative feedback in visual and spatial reversal learning in the rat: behavioural and computational evidence. Psychopharmacology 236(8):2307–2323. 10.1007/s00213-019-05296-y 31218428 10.1007/s00213-019-05296-y PMC6695374

[CR8] Altschul D, Bohn M, Canteloup C, Ebel SJ, Hanus D, Hernandez-Aguilar RA, Joly M, Keupp S, Petkov C, Llorente M, O’Madagain C, Proctor D, Rodrigo AM, Sutherland K, Szabelska A, Taylor D, Völter CJ, Wiggenhauser NG, Primates M (2021) Collaboration and Open Science Initiatives in Primate Research. In Primate Cognitive Studies. 10.31219/osf.io/7c93a

[CR9] Arce W, Stevens JR (2022) A precise dispenser design for canine cognition research. J Open Hardw. 10.5334/joh.41

[CR10] Aria M, Alterisio A, Scandurra A, Pinelli C, D’Aniello B (2021) The scholar’s best friend: research trends in dog cognitive and behavioral studies. Anim Cogn 24(3):541–553. 10.1007/s10071-020-01448-2 33219880 10.1007/s10071-020-01448-2 PMC8128826

[CR11] Ashton RL, De Lillo C (2011) Association, inhibition, and object permanence in dogs’ (*Canis familiaris*) spatial search. J Comp Psychol 125(2):194–206. 10.1037/a0022584 21604853 10.1037/a0022584

[CR12] Attalla D, Schatz A, Stumpenhorst K, Winter Y (2024) Cognitive training of mice attenuates age-related decline in associative learning and behavioral flexibility. Front Behav Neurosci. 10.3389/fnbeh.2024.1326501 38549621 10.3389/fnbeh.2024.1326501 PMC10976437

[CR13] Aust U, Range F, Steurer M, Huber L (2008) Inferential reasoning by exclusion in pigeons, dogs, and humans. Anim Cogn 11(4):587–597. 10.1007/s10071-008-0149-0 18309524 10.1007/s10071-008-0149-0

[CR14] Autier-Dérian D, Deputte BL, Chalvet-Monfray K, Coulon M, Mounier L (2013) Visual discrimination of species in dogs (*Canis familiaris*). Anim Cogn 16(4):637–651. 10.1007/s10071-013-0600-8 23404258 10.1007/s10071-013-0600-8

[CR15] Badampudi D, Wohlin C, Petersen K (2015) Experiences from using snowballing and database searches in systematic literature studies. Proc 19th Int Conf Eval Asses Softw Eng 1–10. 10.1145/2745802.2745818

[CR16] Barber ALA, Randi D, Müller CA, Huber L (2016) The processing of human emotional faces by pet and lab dogs: evidence for lateralization and experience effects. PLoS One 11(4):e0152393. 10.1371/journal.pone.0152393 27074009 10.1371/journal.pone.0152393 PMC4830442

[CR17] Barnard S, Wells DL, Milligan ADS, Arnott G, Hepper PG (2018) Personality traits affecting judgement bias task performance in dogs (*Canis familiaris*). Sci Rep 8(1):6660. 10.1038/s41598-018-25224-y 29703989 10.1038/s41598-018-25224-y PMC5924375

[CR18] Bensky MK, Gosling SD, Sinn DL (2013) The World from a Dog’s Point of View. Advances in the Study of Behavior, vol 45. Elsevier, pp 209–406. 10.1016/B978-0-12-407186-5.00005-7

[CR19] Beraldo FH, Palmer D, Memar S, Wasserman DI, Lee W-J, Liang S, Creighton SD, Kolisnyk B, Cowan MF, Mels J, Masood TS, Fodor C, Al-Onaizi MA, Bartha R, Gee T, Saksida LM, Bussey TJ, Strother SS, Prado VF, Winters BD, Prado MA (2019) MouseBytes, an open-access high-throughput pipeline and database for rodent touchscreen-based cognitive assessment. Elife 8:e49630. 10.7554/eLife.49630 31825307 10.7554/eLife.49630 PMC6934379

[CR20] Bernstein-Kurtycz LM, Vonk J, Carroscia JM, Koester DC, Snyder RJ, Willis MA, Lukas KE (2024) Lack of reinforcement is hard to bear: assessing judgment bias in grizzly bears (*Ursus arctos horribilis*). J Appl Anim Welf Sci 27(3):575–588. 10.1080/10888705.2024.2315042 38363302 10.1080/10888705.2024.2315042

[CR21] Bi X, Li Y, Zhai S (2013) FFitts law: Modeling finger touch with fitts’ law. Proc SIGCHI Conf Hum Factors Comput Syst 1363–1372. 10.1145/2470654.2466180

[CR22] Blaisdell AP, Schroeder JE, Fast CD (2018) Spatial integration during performance in pigeons. Behav Process 154:73–80. 10.1016/j.beproc.2017.12.012 10.1016/j.beproc.2017.12.012 PMC6013331 29274761

[CR23] Booth A (2016) Searching for qualitative research for inclusion in systematic reviews: a structured methodological review. Syst Rev 5(1):74. 10.1186/s13643-016-0249-x 27145932 10.1186/s13643-016-0249-x PMC4855695

[CR24] Bray EE, MacLean EL, Hare BA (2014) Context specificity of inhibitory control in dogs. Anim Cogn 17(1):15–31. 10.1007/s10071-013-0633-z 23584618 10.1007/s10071-013-0633-z PMC4154138

[CR25] Bray EE, MacLean EL, Hare BA (2015) Increasing arousal enhances inhibitory control in calm but not excitable dogs. Anim Cogn 18(6):1317–1329. 10.1007/s10071-015-0901-1 26169659 10.1007/s10071-015-0901-1 PMC4609265

[CR26] Brucks D, Marshall-Pescini S, Wallis LJ, Huber L, Range F (2017) Measures of Dogs’ inhibitory control abilities do not correlate across tasks. Front Psychol 8:849. 10.3389/fpsyg.2017.00849 28596749 10.3389/fpsyg.2017.00849 PMC5443147

[CR27] Brucks D, Soliani M, Range F, Marshall-Pescini S (2017) Reward type and behavioural patterns predict dogs’ success in a delay of gratification paradigm. Sci Rep 7(1):1. 10.1038/srep42459 28272409 10.1038/srep42459 PMC5341119

[CR228] Bunford N, Csibra B, Peták C, Ferdinandy B, Miklósi Á, Gácsi M (2019) Associations among behavioral inhibition and owner-rated attention,hyperactivity/impulsivity, and personality in the domestic dog (Canis familiaris). J Comp Psychol 133(2):233–243. 10.1037/com0000151 30394783 10.1037/com0000151

[CR28] Burton B (2020) Does Clicker Training Lead to Faster Acquisition of Behavior for Dog Owners? Theses and Dissertations. https://academicworks.cuny.edu/hc_sas_etds/528

[CR29] Buscher N, Ojeda A, Francoeur M, Hulyalkar S, Claros C, Tang T, Terry A, Gupta A, Fakhraei L, Ramanathan DS (2020) Open-source raspberry Pi-based operant box for translational behavioral testing in rodents. J Neurosci Methods 342:108761. 10.1016/j.jneumeth.2020.108761 32479970 10.1016/j.jneumeth.2020.108761

[CR30] Butler JL, Kennerley SW (2019) Mymou: a low-cost, wireless touchscreen system for automated training of nonhuman primates. Behav Res Methods 51(6):2559–2572. 10.3758/s13428-018-1109-5 30187433 10.3758/s13428-018-1109-5 PMC6877703

[CR31] Byers-Heinlein K, Bergmann C, Davies C, Frank MC, Hamlin JK, Kline M, Kominsky JF, Kosie JE, Lew-Williams C, Liu L, Mastroberardino M, Singh L, Waddell CPG, Zettersten M, Soderstrom M (2020) Building a collaborative psychological science: lessons learned from ManyBabies 1. Can Psychol / Psychologie Canadienne 61(4):349–363. 10.1037/cap0000216 34219905 10.1037/cap0000216 PMC8244655

[CR33] Byosiere S-E, Chouinard PA, Howell TJ, Bennett PC (2018a) What do dogs (*Canis familiaris*) see? A review of vision in dogs and implications for cognition research. Psychon Bull Rev 25(5):1798–1813. 10.3758/s13423-017-1404-7 29143248 10.3758/s13423-017-1404-7

[CR35] Byosiere S-E, Chouinard PA, Howell TJ, Bennett PC (2019a) Illusory contour perception in domestic dogs. Psychon Bull Rev 26(5):1641–1649. 10.3758/s13423-019-01661-2 31485909 10.3758/s13423-019-01661-2

[CR36] Byosiere S-E, Chouinard PA, Howell TJ, Bennett PC (2019b) The effects of physical luminance on colour discrimination in dogs: A cautionary tale. Appl Anim Behav Sci 212:58–65. 10.1016/j.applanim.2019.01.004

[CR32] Byosiere S-E, Feng LC, Chouinard PA, Howell TJ, Bennett PC (2017) Relational concept learning in domestic dogs: performance on a two-choice size discrimination task generalises to novel stimuli. Behav Process 145:93–101. 10.1016/j.beproc.2017.10.009 10.1016/j.beproc.2017.10.009 29056526

[CR34] Byosiere S-E, Feng LC, Wuister J, Chouinard PA (2018) Do dogs demonstrate susceptibility to a vertically presented Ponzo illusion? Anim Behav Cogn 5(3):254–267. 10.26451/abc.05.03.01.2018

[CR37] Byrne C, Zeagler C, Freil L, Rapoport A, Jackson MM (2018) Dogs using touchscreens in the home: A case study for assistance dogs operating emergency notification systems. Proc Fifth Int Conf Animal-Comput Int 1–10. 10.1145/3295598.3295610

[CR38] Cabrera-Moreno J, Jeanson L, Jeschke M, Calapai A (2022) Group-based, autonomous, individualized training and testing of long-tailed macaques (*Macaca fascicularis*) in their home enclosure to a visuo-acoustic discrimination task. Front Psychol. 10.3389/fpsyg.2022.1047242 36524199 10.3389/fpsyg.2022.1047242 PMC9745322

[CR39] Canori C, Biffi E, Gaggia L, Iuliano B, Valsecchi P (2025) Do looks matter? Investigating facial expressions and intraspecific communication across different dog morphotypes. Appl Anim Behav Sci 291:106720. 10.1016/j.applanim.2025.106720

[CR230] Chapagain D, Virányi Z, Huber L, Serra J, Schoesswender J, Range F (2018) Effect of age and dietary intervention on discrimination learning in pet dogs. Frontiers in Psychology, 9. 10.3389/fpsyg2018.02217 10.3389/fpsyg.2018.02217 PMC6246696 30487772

[CR40] Chasse RY, Perrino PA, McLeod RM, Altmann GTM, Fitch RH (2023) A novel approach to the assessment of higher-order rule learning in male mice. Learn Memory (Cold Spring Harbor N Y) 30(10):271–277. 10.1101/lm.053771.123 10.1101/lm.053771.123 PMC10561631 37802548

[CR41] Cimarelli G, Schoesswender J, Vitiello R, Huber L, Virányi Z (2021) Partial rewarding during clicker training does not improve naïve dogs’ learning speed and induces a pessimistic-like affective state. Anim Cogn 24(1):107–119. 10.1007/s10071-020-01425-9 32897444 10.1007/s10071-020-01425-9 PMC7829239

[CR42] Clark CCA, Sibbald NJ, Rooney NJ (2020) Search dog handlers show positive bias when scoring their own dog’s performance. Front Vet Sci. 10.3389/fvets.2020.00612 33195498 10.3389/fvets.2020.00612 PMC7533607

[CR43] Coile DC, Pollitz CH, Smith JC (1989) Behavioral determination of critical flicker fusion in dogs. Physiol Behav 45(6):1087–1092. 10.1016/0031-9384(89)90092-9 2813532 10.1016/0031-9384(89)90092-9

[CR44] Coles NA, March DS, Marmolejo-Ramos F, Larsen JT, Arinze NC, Ndukaihe ILG, Willis ML, Foroni F, Reggev N, Mokady A, Forscher PS, Hunter JF, Kaminski G, Yüvrük E, Kapucu A, Nagy T, Hajdu N, Tejada J, Freitag RMK, Liuzza MT (2022) A multi-lab test of the facial feedback hypothesis by the Many Smiles Collaboration. Nat Hum Behav 6(12):1731–1742. 10.1038/s41562-022-01458-9 36266452 10.1038/s41562-022-01458-9

[CR45] Craig M, Rand J, Mesch R, Shyan-Norwalt M, Morton J, Flickinger E (2012) Domestic dogs (*Canis familiaris*) and the radial arm maze: spatial memory and serial position effects. J Comp Psychol 126(3):233–242. 10.1037/a0025929 22905996 10.1037/a0025929

[CR46] Crijns E, de Op Beeck H (2019) The visual acuity of rats in touchscreen setups. Vision 4:4. 10.3390/vision4010004 31906140 10.3390/vision4010004 PMC7157561

[CR47] Dale R, Palma-Jacinto S, Marshall-Pescini S, Range F (2019) Wolves, but not dogs, are prosocial in a touch screen task. PLoS One 14(5):e0215444. 10.1371/journal.pone.0215444 31042740 10.1371/journal.pone.0215444 PMC6493736

[CR49] Degrande R, Cornilleau F, Lansade L, Jardat P, Colson V, Calandreau L (2022) Domestic hens succeed at serial reversal learning and perceptual concept generalisation using a new automated touchscreen device. Animal 16(8):100607. 10.1016/j.animal.2022.100607 35963029 10.1016/j.animal.2022.100607

[CR50] Demant H, Ladewig J, Balsby TJS, Dabelsteen T (2011) The effect of frequency and duration of training sessions on acquisition and long-term memory in dogs. Appl Anim Behav Sci 133(3):228–234. 10.1016/j.applanim.2011.05.010

[CR48] De Moor D, Skelton M, MacaqueNet, Amici F, Arlet ME, Balasubramaniam KN, Ballesta S, Berghänel A, Berman CM, Bernstein SK, Bhattacharjee D, Bliss-Moreau E, Brotcorne F, Butovskaya M, Campbell LAD, Carosi M, Chatterjee M, Cooper MA, Cowl VB, Brent LJN (2025) MacaqueNet: advancing comparative behavioural research through large-scale collaboration. J Anim Ecol 94(4):519–534. 10.1111/1365-2656.14223 39934999 10.1111/1365-2656.14223 PMC11962231

[CR51] Devarakonda K, Nguyen KP, Kravitz AV (2015) ROBucket: a low cost operant chamber based on the Arduino microcontroller. Behav Res Methods 48:503–509. 10.3758/s13428-015-0603-2 10.3758/s13428-015-0603-2 26019006

[CR52] Dror S, Miklósi Á, Fugazza C (2024) Dogs with a vocabulary of object labels retain them for at least 2 years. Biol Lett 20(9):20240208. 10.1098/rsbl.2024.0208 39226922 10.1098/rsbl.2024.0208 PMC11371427

[CR53] Dror S, Miklósi Á, Morvai B, Năstase A-S, Fugazza C (2026) Dogs with a large vocabulary of object labels learn new labels by overhearing like 1.5-year-old infants. Science 391(6781):160–163. 10.1126/science.adq5474 41505543 10.1126/science.adq5474

[CR54] Dumont JR, Salewski R, Beraldo F (2020) Critical mass: the rise of a touchscreen technology community for rodent cognitive testing. Genes Brain Behav. 10.1111/gbb.12650 32141694 10.1111/gbb.12650

[CR55] Dumont JR, Salewski R, Beraldo F (2021) Critical mass: the rise of a touchscreen technology community for rodent cognitive testing. Genes Brain Behav 20(1):e12650. 10.1111/gbb.12650 32141694 10.1111/gbb.12650

[CR56] Egelkamp CL, Ross SR (2019) A review of zoo-based cognitive research using touchscreen interfaces. Zoo Biol 38(2):220–235. 10.1002/zoo.21458 30480845 10.1002/zoo.21458

[CR57] Elliott JJ, Miller CT, Hagarman JA, Kelley ST, Tardif SD, Hacker SO, Bettis A (2017) Management of Research Animal Breeding Colonies. Management of Animal Care and Use Programs in Research, Education, and Testing, 2nd edn. CRC 29787204

[CR58] Fagnani J, Barrera G, Carballo F, Bentosela M (2016a) Is previous experience important for inhibitory control? A comparison between shelter and pet dogs in A-not-B and cylinder tasks. Anim Cogn 19(6):1165–1172. 10.1007/s10071-016-1024-z 27541147 10.1007/s10071-016-1024-z

[CR59] Fagnani J, Barrera G, Carballo F, Bentosela M (2016b) Tolerance to delayed reward tasks in social and non-social contexts. Behavioural Proc 130:19–30. 10.1016/j.beproc.2016.06.011 10.1016/j.beproc.2016.06.011 27343621

[CR60] Fagot J, Paleressompoulle D (2009) Automatic testing of cognitive performance in baboons maintained in social groups. Behav Res Methods 41(2):396–404. 10.3758/BRM.41.2.396 19363180 10.3758/BRM.41.2.396

[CR61] Feng LC, Howell TJ, Bennett PC (2016) How clicker training works: comparing reinforcing, marking, and bridging hypotheses. Appl Anim Behav Sci 181:34–40. 10.1016/j.applanim.2016.05.012

[CR62] Fiset S, Beaulieu C, Landry F (2003) Duration of dogs’ (*Canis familiaris*) working memory in search for disappearing objects. Anim Cogn 6(1):1–10. 10.1007/s10071-002-0157-4 12658530 10.1007/s10071-002-0157-4

[CR63] Fitts PM (1954) The information capacity of the human motor system in controlling the amplitude of movement. J Exp Psychol 47(6):381–391. 10.1037/h0055392 13174710

[CR64] Fizet J, Rimele A, Cassel J-C, Kelche C, Meunier H (2017) An autonomous, automated and mobile device to concurrently assess several cognitive functions in group-living non-human primates. Neurobiol Learn Mem 145:45–58. 10.1016/j.nlm.2017.07.013 28774735 10.1016/j.nlm.2017.07.013

[CR65] Fugazza C, Andics A, Magyari L, Dror S, Zempléni A, Miklósi Á (2021) Rapid learning of object names in dogs. Sci Rep 11(1):2222. 10.1038/s41598-021-81699-2 33500506 10.1038/s41598-021-81699-2 PMC7838202

[CR229] Galambos, Á., Petró, E., Nagy, B., Turcsán, B., & Topál, J. (2021). The effects of social and non-social distracting stimuli on dogs with diff erent levels of social competence –Empirical evidence for a canine model of autism. Applied Animal Behaviour Science, 244 , 105451. 10.1016/j.applanim.2021.105451

[CR66] Ganguli M, Lee C-W, Hughes T, Snitz BE, Jakubcak J, Duara R, Chang C-CH (2015) Who wants a free brain scan? Assessing and correcting for recruitment biases in a population-based sMRI pilot study. Brain Imaging Behav 9(2):204–212. 10.1007/s11682-014-9297-9 24573773 10.1007/s11682-014-9297-9 PMC4147027

[CR67] Gao J, Adachi I (2024) Body part categorical matching in chimpanzees (*Pan troglodytes*). Sci Rep 14(1):15896. 10.1038/s41598-024-66829-w 38987277 10.1038/s41598-024-66829-w PMC11236962

[CR68] Gao J, Yamanashi Y, Tanaka M (2025) Touchscreen tasks for cognitive testing in domestic goats (*Capra hircus*): a pilot study using odd-item search training. Animals 15(14):2115. 10.3390/ani15142115 40723578 10.3390/ani15142115 PMC12291992

[CR69] Gazes RP, Diamond RFL, Hope JM, Caillaud D, Stoinski TS, Hampton RR (2017) Spatial representation of magnitude in gorillas and orangutans. Cognition 168:312–319. 10.1016/j.cognition.2017.07.010 28772188 10.1016/j.cognition.2017.07.010

[CR70] Gilchrist RJ, Gunter LM, Anderson SF, Wynne CDL (2021) The click is not the trick: the efficacy of clickers and other reinforcement methods in training naïve dogs to perform new tasks. PeerJ 9:e10881. 10.7717/peerj.10881 33665026 10.7717/peerj.10881 PMC7906040

[CR71] Greenwald AG (1975) Consequences of prejudice against the null hypothesis. Psychol Bull 82(1):1–20. 10.1037/h0076157

[CR72] Griggs DJ, Bloch J, Chavan S, Coubrough KM, Conley W, Morrisroe K, Yazdan-Shahmorad A (2021) Autonomous cage-side system for remote training of non-human primates. J Neurosci Methods 348:108969. 10.1016/j.jneumeth.2020.108969 33039414 10.1016/j.jneumeth.2020.108969 PMC8384435

[CR73] Group PRISMA-P, Moher D, Shamseer L, Clarke M, Ghersi D, Liberati A, Petticrew M, Shekelle P, Stewart LA (2015) Preferred reporting items for systematic review and meta-analysis protocols (PRISMA-P) 2015 statement. Syst Reviews 4(1):1. 10.1186/2046-4053-4-1 10.1186/2046-4053-4-1 PMC4320440 25554246

[CR74] Gupta S (2024) AI Applications in Animal Behavior Analysis and Welfare. Agriculture 4.0. CRC

[CR75] Hall LS, Boxall J (2024) The laboratory dog. In: Golledge H, Richardson C (eds) The UFAW Handbook on the Care and Management of Laboratory and Other Research Animals, 1st ed. Wiley, pp 518–545. 10.1002/9781119555278.ch30

[CR76] Hall NJ, Smith DW, Wynne CDL (2013) Training domestic dogs (*Canis lupus familiaris*) on a novel discrete trials odor-detection task. Learn Motiv 44(4):218–228. 10.1016/j.lmot.2013.02.004

[CR77] Handley K, Hazel S, Fountain J, Fernandez EJ (2023) Comparing trial-and-error to errorless learning procedures in training pet dogs a visual discrimination. Learn Motiv 84:101944. 10.1016/j.lmot.2023.101944

[CR78] Harrison RA, Mohr T, van de Waal E (2023) Lab cognition going wild: implementing a new portable touchscreen system in vervet monkeys. J Anim Ecol 92(8):1545–1559. 10.1111/1365-2656.13857 36635850 10.1111/1365-2656.13857

[CR79] Head E, Mehta R, Hartley J, Kameka M, Cummings BJ, Cotman CW, Ruehl WW, Milgram NW (1995) Spatial learning and memory as a function of age in the dog. Behav Neurosci 109(5):851–858. 10.1037/0735-7044.109.5.851 8554710 10.1037//0735-7044.109.5.851

[CR80] Heinrich DDU, Pouca V, Brown C, Huveneers C (2020) Effects of reward magnitude and training frequency on the learning rates and memory retention of the Port Jackson shark *Heterodontus portusjacksoni*. Anim Cogn 23(5):939–949. 10.1007/s10071-020-01402-2 32524291 10.1007/s10071-020-01402-2

[CR81] Higgins J, Thomas J (2019) Cochrane Handbook for Systematic Reviews of Interventions., 2nd ed. Wiley-Blackwell

[CR82] Hirskyj-Douglas I, Read JC, Cassidy B (2017) A dog centred approach to the analysis of dogs’ interactions with media on TV screens. Int J Hum Comput Stud 98:208–220. 10.1016/j.ijhcs.2016.05.007

[CR83] Holman L, Head ML, Lanfear R, Jennions MD (2015) Evidence of experimental bias in the life sciences: why we need blind data recording. PLoS Biol 13(7):e1002190. 10.1371/journal.pbio.1002190 26154287 10.1371/journal.pbio.1002190 PMC4496034

[CR84] Holzbach RL (1978) Rater bias in performance ratings: superior, self-, and peer ratings. J Appl Psychol 63(5):579–588. 10.1037/0021-9010.63.5.579

[CR85] Hopper LM, Allritz M, Egelkamp CL, Huskisson SM, Jacobson SL, Leinwand JG, Ross SR (2021) A comparative perspective on three primate species’ responses to a pictorial emotional Stroop task. Animals 11(3):3. 10.3390/ani11030588 10.3390/ani11030588 PMC7995981 33668170

[CR86] Horner AE, Heath CJ, Hvoslef-Eide M, Kent BA, Kim CH, Nilsson SRO, Alsiö J, Oomen CA, Holmes A, Saksida LM, Bussey TJ (2013) The touchscreen operant platform for testing learning and memory in rats and mice. Nat Protoc 8(10):1961–1984. 10.1038/nprot.2013.122 24051959 10.1038/nprot.2013.122 PMC3914026

[CR87] Hoyt WT (2000) Rater bias in psychological research: when is it a problem and what can we do about it? Psychol Methods 5(1):64–86. 10.1037/1082-989X.5.1.64 10937323 10.1037/1082-989x.5.1.64

[CR88] Huber L, Apfalter W, Steurer M, Prossinger H (2005) A new learning paradigm elicits fast visual discrimination in pigeons. J Exp Psychol Anim Behav Process 31(2):237–246. 10.1037/0097-7403.31.2.237 15839779 10.1037/0097-7403.31.2.237

[CR89] Huskisson SM, Jacobson SL, Egelkamp CL, Ross SR, Hopper LM (2020) Using a touchscreen paradigm to evaluate food preferences and response to novel photographic stimuli of food in three primate species (*Gorilla gorilla gorilla*, *Pan troglodytes*, and *Macaca fuscata*). Int J Primatol 41(1):5–23. 10.1007/s10764-020-00131-0

[CR90] Huskisson SM, Ross SR, Hopper LM (2021) Do zoo visitors induce attentional bias effects in primates completing cognitive tasks? Anim Cogn 24(3):645–653. 10.1007/s10071-020-01445-5 33156406 10.1007/s10071-020-01445-5

[CR91] Hutchins EL, Hollan JD, Norman DA (1985) Direct manipulation interfaces. Hum Comput Interact 1(4):311–338. 10.1207/s15327051hci0104_2

[CR92] Ichikawa Y, Kanemaki N, Kanai K (2024) Breed-specific skull morphology reveals insights into canine optic chiasm positioning and orbital structure through 3D CT scan analysis. Animals 14(2):197. 10.3390/ani14020197 38254367 10.3390/ani14020197 PMC10812588

[CR93] International Brain Laboratory, International Brain Laboratory (2017) An international laboratory for systems and computational neuroscience. Neuron 96(6):1213–1218. 10.1016/j.neuron.2017.12.013 29268092 10.1016/j.neuron.2017.12.013 PMC5752703

[CR94] Jackson MM, Byrne C, Freil L, Valentin G, Zuerndorfer J, Zeagler C, Logas J, Gilliland S, Rapoport A, Sun S, Peet D, Lau A, Han X, Alcaidinho J, Starner T (2018) Technology for working dogs. Proc Fifth Int Conf Animal-Comput Int 1–5. 10.1145/3295598.3295615

[CR95] Johnen D, Heuwieser W, Fischer-Tenhagen C (2017) An approach to identify bias in scent detection dog testing. Appl Anim Behav Sci 189:1–12. 10.1016/j.applanim.2017.01.001

[CR96] Kane GA, Lopes G, Saunders JL, Mathis A, Mathis MW (2020) Real-time, low-latency closed-loop feedback using markerless posture tracking. Elife 9:e61909. 10.7554/eLife.61909 33289631 10.7554/eLife.61909 PMC7781595

[CR97] Kangas BD, Bergman J (2017) Touchscreen technology in the study of cognition-related behavior. Behav Pharmacol 28:623–629. 10.1097/fbp.0000000000000356 29064843 10.1097/FBP.0000000000000356 PMC5687822

[CR98] Kanizsár O, Mongillo P, Battaglini L, Campana G, Marinelli L (2017) Dogs are not better than humans at detecting coherent motion. Sci Rep 7(1):11259. 10.1038/s41598-017-11864-z 28900293 10.1038/s41598-017-11864-z PMC5595918

[CR99] Karl S, Boch M, Zamansky A, Van Der Linden D, Wagner IC, Völter CJ, Lamm C, Huber L (2020) Exploring the dog–human relationship by combining fMRI, eye-tracking and behavioural measures. Sci Rep 10(1):22273. 10.1038/s41598-020-79247-5 33335230 10.1038/s41598-020-79247-5 PMC7747637

[CR100] Keep B, Zulch HE, Wilkinson A (2018) Truth is in the eye of the beholder: perception of the Müller-Lyer illusion in dogs. Learn Behav 46(4):501–512. 10.3758/s13420-018-0344-z 30187301 10.3758/s13420-018-0344-z PMC6276079

[CR101] Kenawell A, Crossen A, Hamann K, Nadler S, Simpson C, Winship K, Highfill L (2025) Evaluating a four-button computerized gaming system for cognitive engagement in dogs. Learn Behav. 10.3758/s13420-025-00692-1 41186871 10.3758/s13420-025-00692-1

[CR102] Khan A, Khusro S (2019) Blind-friendly user interfaces – a pilot study on improving the accessibility of touchscreen interfaces. Multimedia Tools Appl 78(13):17495–17519. 10.1007/s11042-018-7094-y

[CR103] Kim CH, Hvoslef-Eide M, Nilsson SRO, Johnson MR, Herbert BR, Robbins TW, Saksida LM, Bussey TJ, Mar AC (2015) The continuous performance test (rCPT) for mice: a novel operant touchscreen test of attentional function. Psychopharmacology 232(21–22):3947–3966. 10.1007/s00213-015-4081-0 26415954 10.1007/s00213-015-4081-0 PMC4600477

[CR104] Kleinberger R, Cunha J, McMahon M, Hirskyj-Douglas I (2024) No More Angry Birds: Investigating Touchscreen Ergonomics to Improve Tablet-Based Enrichment for Parrots. Proc 2024 CHI Conf Human Fact Comput Syst 1–16. 10.1145/3613904.3642119

[CR105] Kravitz A, Laubach M (2024) Unleashing the power of DIY innovation in behavioral neuroscience. The Transmitter

[CR106] Krichbaum S, Smith JG, Lazarowski L, Katz JS (2021) Controlling for dogs’ (*Canis familiaris*) use of nonmnemonic strategies in a spatial working memory task. J Experimental Psychology: Anim Learn Cognition 47(3):364–370. 10.1037/xan0000293 10.1037/xan0000293 34618534

[CR107] Lambert M, Farrar B, Garcia-Pelegrin E, Reber S, Miller R (2022) ManyBirds: a multi-site collaborative Open Science approach to avian cognition and behavior research. Anim Behav Cogn 9(1):133–152. 10.26451/abc.09.01.11.2022

[CR108] Langbein J, Moreno-Zambrano M, Siebert K (2023) How do goats “read” 2D-images of familiar and unfamiliar conspecifics? Front Psychol. 10.3389/fpsyg.2023.1089566 37275711 10.3389/fpsyg.2023.1089566 PMC10236219

[CR109] Laude JR, Pattison KF, Rayburn-Reeves RM, Michler DM, Zentall TR (2016) Who are the real bird brains? Qualitative differences in behavioral flexibility between dogs (*Canis familiaris*) and pigeons (*Columba livia*). Anim Cogn 19(1):163–169. 10.1007/s10071-015-0923-8 26364290 10.1007/s10071-015-0923-8

[CR110] Lauer J, Zhou M, Ye S, Menegas W, Schneider S, Nath T, Rahman MM, Di Santo V, Soberanes D, Feng G, Murthy VN, Lauder G, Dulac C, Mathis MW, Mathis A (2022) Multi-animal pose estimation, identification and tracking with DeepLabCut. Nat Methods 19(4):496–504. 10.1038/s41592-022-01443-0 35414125 10.1038/s41592-022-01443-0 PMC9007739

[CR111] Lazarowski L, Rogers B, Collins-Pisano C, Krichbaum S, Handley M, Smith JG, Waggoner P (2025) Effectiveness of marker training for detection dogs. Front Vet Sci. 10.3389/fvets.2025.1538452 40061907 10.3389/fvets.2025.1538452 PMC11885294

[CR112] Lazzaroni M, Range F, Bernasconi L, Darc L, Holtsch M, Massimei R, Rao A, Marshall-Pescini S (2019) The role of life experience in affecting persistence: a comparative study between free-ranging dogs, pet dogs and captive pack dogs. PLoS One 14(4):e0214806. 10.1371/journal.pone.0214806 30995264 10.1371/journal.pone.0214806 PMC6469757

[CR113] Lee J-H, Spence C (2008) Feeling what you hear: task-irrelevant sounds modulate tactile perception delivered via a touch screen. J Multimodal User Interfaces 2(3):145–156. 10.1007/s12193-009-0014-8

[CR115] Liberati A, Altman DG, Tetzlaff J, Mulrow C, Gøtzsche PC, Ioannidis JPA, Clarke M, Devereaux PJ, Kleijnen J, Moher D (2009) The PRISMA statement for reporting systematic reviews and meta-analyses of studies that evaluate health care interventions: explanation and elaboration. PLoS Med 6(7):e1000100. 10.1371/journal.pmed.1000100 19621070 10.1371/journal.pmed.1000100 PMC2707010

[CR116] Lind O, Milton I, Andersson E, Jensen P, Roth LSV (2017) High visual acuity revealed in dogs. PLoS One 12(12):e0188557. 10.1371/journal.pone.0188557 29206864 10.1371/journal.pone.0188557 PMC5716585

[CR114] Li Z, Han J, Ji M, Tang L-A, Yu Y, Ding B, Lee J-G, Kays R (2011) Movemine: mining moving object data for discovery of animal movement patterns. ACM Trans Intell Syst Technol 2(4):37:1–37:32. 10.1145/1989734.1989741

[CR118] Lõoke M, Kanizsàr O, Battaglini L, Guerineau C, Mongillo P, Marinelli L (2020) Are dogs good at spotting movement? Velocity thresholds of motion detection in *Canis familiaris*. Curr Zool 66(6):699–701. 10.1093/cz/zoaa044 33391371 10.1093/cz/zoaa044 PMC7769578

[CR117] Lonardo L, Versace E, Huber L (2022) Recognition of rotated objects and cognitive offloading in dogs. iScience. 10.1016/j.isci.2022.103820 35198883 10.1016/j.isci.2022.103820 PMC8841888

[CR119] Lopes G, Monteiro P (2021) New open-source tools: using Bonsai for behavioral tracking and closed-loop experiments. Front Behav Neurosci. 10.3389/fnbeh.2021.647640 33867952 10.3389/fnbeh.2021.647640 PMC8044343

[CR120] Lorenzi E, Vallortigara G (2021) Evolutionary and Neural Bases of the Sense of Animacy. In: Kaufman AB, Call J, Kaufman JC (eds) The Cambridge Handbook of Animal Cognition, 1st ed. Cambridge University Press, pp 295–321. 10.1017/9781108564113.017

[CR121] Loyant L, Waller BM, Micheletta J, Joly M (2022) Validation of a battery of inhibitory control tasks reveals a multifaceted structure in non-human primates. PeerJ 10:e12863. 10.7717/peerj.12863 35186469 10.7717/peerj.12863 PMC8840138

[CR122] Lucca K, Yuen F, Wang Y, Alessandroni N, Allison O, Alvarez M, Axelsson EL, Baumer J, Baumgartner HA, Bertels J, Bhavsar M, Byers-Heinlein K, Capelier-Mourguy A, Chijiiwa H, Chin CS-S, Christner N, Cirelli LK, Corbit J, Daum MM, Hamlin JK (2025) Infants’ Social Evaluation of Helpers and Hinderers: A Large-Scale, Multi-Lab, Coordinated Replication Study. Dev Sci 28(1):e13581. 10.1111/desc.13581 39600132 10.1111/desc.13581

[CR123] MacLean EL, Hare B, Nunn CL, Addessi E, Amici F, Anderson RC, Aureli F, Baker JM, Bania AE, Barnard AM, Boogert NJ, Brannon EM, Bray EE, Bray J, Brent LJN, Burkart JM, Call J, Cantlon JF, Cheke LG, Zhao Y (2014) The evolution of self-control. Proc National Acad Sci 111(20). 10.1073/pnas.1323533111 10.1073/pnas.1323533111 PMC4034204 24753565

[CR124] Malassis R, Seed AM (2023) Do they know or just do it? Investigating implicit and explicit sequence learning by capuchin monkeys, human adults and children. Conscious Cogn 114:103557. 10.1016/j.concog.2023.103557 37579700 10.1016/j.concog.2023.103557

[CR125] ManyDogs, Espinosa J, Bray EE, Buchsbaum D, Byosiere S, Byrne M, Freeman M, Gnanadesikan GE, Guran C-NA, Horschler D, Huber L, Johnston AM, MacLean EL, Pelgrim MH, Santos LR, Silver ZA, Stevens JR, Völter CJ, Zipperling LML, Zylberfuden SG (2021) ManyDogs 1: A Multi-Lab Replication Study of Dogs’ Pointing Comprehension. PsyArXiv (OSF Preprints). 10.31234/osf.io/f86jq

[CR126] ManyGoats, Battini M, Berthelot M, Daros R, Freeman MS, Nawroth C, Neave H, Ventura B, Zobel G (2024) ManyGoats1—Assessing the impact of human attention on avoidance distance in goats [Stage 1 Registered Report]. OSF. 10.31219/osf.io/hb2sn

[CR127] ManyPrimates, Aguenounon G, Allritz M, Altschul D, Ballesta S, Beaud A, Bohn M, Bornbusch S, Brandão A, Brooks J, Bugnyar T, Burkart J, Bustamante L, Call J, Canteloup C, Cao C, Caspar K, Da Silva D, De Sousa A, Zablocki-Thomas P (2020) The Evolution of Primate Short-Term Memory. Anim Behav Cognition 9(4):428–516. 10.26451/abc.09.04.06.2022

[CR128] Marquardt K, Sigdel R, Brigman JL (2017) Touch-screen visual reversal learning is mediated by value encoding and signal propagation in the orbitofrontal cortex. Neurobiol Learn Mem 139:179–188. 10.1016/j.nlm.2017.01.006 28111339 10.1016/j.nlm.2017.01.006 PMC5372695

[CR129] Marshall-Pescini S, Virányi Z, Range F (2015) The effect of domestication on inhibitory control: wolves and dogs compared. PLoS One 10(2):e0118469. 10.1371/journal.pone.0118469 25714840 10.1371/journal.pone.0118469 PMC4340790

[CR130] Martin CF, Muramatsu A, Matsuzawa T (2022) Apex and apetouch: development of a portable touchscreen system and software for primates at zoos. Animals 12(13):1660. 10.3390/ani12131660 35804559 10.3390/ani12131660 PMC9265006

[CR131] Mathis A, Mamidanna P, Cury KM, Abe T, Murthy VN, Mathis MW, Bethge M (2018) Deeplabcut: markerless pose estimation of user-defined body parts with deep learning. Nat Neurosci 21(9):1281–1289. 10.1038/s41593-018-0209-y 30127430 10.1038/s41593-018-0209-y

[CR132] McEwen ES, Allritz M, Call J, Koopman SE, Rapport Munro E, Bottero Cantuarias CJ, Menzel CR, Dolins FL, Janmaat KRL, Schweller K (2025) Training primates to forage in virtual 3D environments. Behav Process 224:105126. 10.1016/j.beproc.2024.105126 10.1016/j.beproc.2024.105126 39667534

[CR133] Menaker T, Zamansky A, van der Linden D, Kaplun D, Sinitica A, Karl S, Huber L (2021) Towards a Methodology for Data-Driven Automatic Analysis of Animal Behavioral Patterns. Proc Seven Int Conf Animal-Comput Int 1–6. 10.1145/3446002.3446126

[CR134] Mendes F, Brino A, Goulart PRK, Galvão O, Ventura DSF, Miquilini L, Brito F, Souza GS (2024) Investigation of preference for local and global processing of Capuchin-monkeys (*Sapajus* spp.) in shape discrimination of mosaic arrangements. PLOS ONE 19(5):e0303562. 10.1371/journal.pone.0303562 38809944 10.1371/journal.pone.0303562 PMC11135723

[CR135] Meyer I, Ladewig J (2008) The relationship between number of training sessions per week and learning in dogs. Appl Anim Behav Sci 111(3):311–320. 10.1016/j.applanim.2007.06.016

[CR136] Michelson L, Mannarino A, Marchione K, Kazdin AE, Costello A (1985) Expectancy bias in behavioral observations of therapeutic outcome: an experimental analysis of treatment and halo effects. Behav Res Ther 23(4):407–414. 10.1016/0005-7967(85)90168-8 4026770 10.1016/0005-7967(85)90168-8

[CR137] Miletto Petrazzini ME, Wynne CDL (2016) What counts for dogs (*Canis lupus familiaris*) in a quantity discrimination task? Behav Process 122:90–97. 10.1016/j.beproc.2015.11.013 10.1016/j.beproc.2015.11.013 26601897

[CR138] Milgram NW (2003) Cognitive experience and its effect on age-dependent cognitive decline in Beagle dogs. Neurochem Res 28(11):1677–1682. 10.1023/A:1026009005108 14584821 10.1023/a:1026009005108

[CR139] Milgram NW, Head E, Weiner E, Thomas E (1994) Cognitive functions and aging in the dog: acquisition of nonspatial visual tasks. Behav Neurosci 108(1):57–68. 10.1037/0735-7044.108.1.57 10.1037//0735-7044.108.1.57 8192851

[CR142] Miller HC, Rayburn-Reeves R, Zentall TR (2009) What do dogs know about hidden objects? Behav Process 81(3):439–446. 10.1016/j.beproc.2009.03.018 10.1016/j.beproc.2009.03.018 PMC2696049 19520244

[CR141] Miller LE, Stewart ME (2011) The blind leading the blind: use and misuse of blinding in randomized controlled trials. Contemp Clin Trials 32(2):240–243. 10.1016/j.cct.2010.11.004 21070890 10.1016/j.cct.2010.11.004

[CR140] Miller PE, Murphy CJ (1995) Vision in dogs. J Am Vet Med Assoc 207(12):1623–1634 7493905

[CR147] Müller CA, Schmitt K, Barber ALA, Huber L (2015) Dogs can discriminate emotional expressions of human faces. Curr Biol 25:601–605. 10.1016/j.cub.2014.12.055 25683806 10.1016/j.cub.2014.12.055

[CR143] Mluba HS, Atif O, Lee J, Park D, Chung Y (2024) Pattern mining-based pig behavior analysis for health and welfare monitoring. Sensors 24(7):7. 10.3390/s24072185 10.3390/s24072185 PMC11013991 38610396

[CR145] Mongillo P, Eatherington C, Lõoke M, Marinelli L (2021) I know a dog when i see one: dogs (*Canis familiaris*) recognize dogs from videos. Anim Cogn 24(5):969–979. 10.1007/s10071-021-01470-y 33740148 10.1007/s10071-021-01470-y PMC8360863

[CR144] Mongillo P, Pitteri E, Sambugaro P, Carnier P, Marinelli L (2017) Global bias reliability in dogs (*Canis familiaris*). Anim Cogn 20(2):257–265. 10.1007/s10071-016-1044-8 27738840 10.1007/s10071-016-1044-8

[CR146] Mueller-Paul J, Wilkinson A, Aust U, Steurer M, Hall G, Huber L (2014) Touchscreen performance and knowledge transfer in the red-footed tortoise (*Chelonoidis carbonaria*). Behav Process 106:187–192. 10.1016/j.beproc.2014.06.003 10.1016/j.beproc.2014.06.003 24946312

[CR148] Muramatsu A, Matsuzawa T (2023) Sequence order in the range 1 to 19 by chimpanzees on a touchscreen task: processing two-digit Arabic numerals. Animals 13(5):5. 10.3390/ani13050774 10.3390/ani13050774 PMC10000109 36899632

[CR149] Nasrini J, Hampton RR (2024) No evidence of real-world equivalence in chickens (*Gallus gallus domesticus*) categorizing visually diverse images of natural stimuli presented on LCD monitors. Learn Behav. 10.3758/s13420-024-00623-6 38267730 10.3758/s13420-024-00623-6 PMC11266530

[CR150] Nath T, Mathis A, Chen AC, Patel A, Bethge M, Mathis MW (2019) Using deeplabcut for 3D markerless pose estimation across species and behaviors. Nat Protoc 14(7):2152–2176. 10.1038/s41596-019-0176-0 31227823 10.1038/s41596-019-0176-0

[CR151] Neitz J, Geist T, Jacobs GH (1989) Color vision in the dog. Vis Neurosci 3(2):119–125. 10.1017/s0952523800004430 2487095 10.1017/s0952523800004430

[CR152] Norman DA (1999) Affordance, conventions, and design. Interactions 6(3):38–43. 10.1145/301153.301168

[CR155] Odland AU, Sandahl R, Andreasen JT (2021) Sequential reversal learning: a new touchscreen schedule for assessing cognitive flexibility in mice. Psychopharmacology 238(2):383–397. 10.1007/s00213-020-05687-6 33123820 10.1007/s00213-020-05687-6

[CR153] O’Hara M, Mioduszewska B, von Bayern A, Auersperg A, Bugnyar T, Wilkinson A, Huber L, Gajdon GK (2017) The temporal dependence of exploration on neotic style in birds. Sci Rep. 10.1038/s41598-017-04751-0 28684773 10.1038/s41598-017-04751-0 PMC5500574

[CR154] O’Leary JD, O’Leary OF, Cryan JF, Nolan YM (2018) A low-cost touchscreen operant chamber using a Raspberry Pi^™^. Behav Res Methods 50:2523–2530. 10.3758/s13428-018-1030-y 29520633 10.3758/s13428-018-1030-y

[CR156] Open Science Collaboration (2015) Estimating the reproducibility of psychological science. Science 349(6251):aac4716. 10.1126/science.aac4716 26315443 10.1126/science.aac4716

[CR157] Orphanides AK, Nam CS (2017) Touchscreen interfaces in context: a systematic review of research into touchscreens across settings, populations, and implementations. Appl Ergon 61:116–143. 10.1016/j.apergo.2017.01.013 28237011 10.1016/j.apergo.2017.01.013

[CR158] Oswald LM, Wand GS, Zhu S, Selby V (2013) Volunteerism and self-selection bias in human positron emission tomography neuroimaging research. Brain Imaging Behav 7(2):163–176. 10.1007/s11682-012-9210-3 23196924 10.1007/s11682-012-9210-3 PMC3594082

[CR159] Oxley JA, Santa K, Meyer G, Westgarth C (2022) A Systematic Scoping Review of Human-Dog Interactions in Virtual and Augmented Reality: The Use of Virtual Dog Models and Immersive Equipment. Front Virtual Real 3. 10.3389/frvir.2022.782023

[CR160] Palmer D, Dumont JR, Dexter TD, Prado MAM, Finger E, Bussey TJ, Saksida LM (2021) Touchscreen cognitive testing: cross-species translation and co-clinical trials in neurodegenerative and neuropsychiatric disease. Neurobiol Learn Mem 182:107443. 10.1016/j.nlm.2021.107443 33895351 10.1016/j.nlm.2021.107443

[CR161] Parhi P, Karlson AK, Bederson BB (2006) Target size study for one-handed thumb use on small touchscreen devices. Proc 8th Conf Human-Comput Int Mobile Dev Ser 203–210. 10.1145/1152215.1152260

[CR162] Pearce J, Qian J-Y (2022) Economic impact of DIY home manufacturing of consumer products with low-cost 3D printing from free and open source designs. European Journal of Social Impact and Circular Economy 3(2):2. 10.13135/2704-9906/6508

[CR163] Perdue B (2016) The effect of computerized testing on sun bear behavior and enrichment preferences. Behav Sci (Basel) 6:19. 10.3390/bs6040019 27669314 10.3390/bs6040019 PMC5197932

[CR164] Perdue BM, Clay AW, Gaalema DE, Maple TL, Stoinski TS (2012) Technology at the zoo: the influence of a touchscreen computer on orangutans and zoo visitors. Zoo Biol 31(1):27–39. 10.1002/zoo.20378 21319214 10.1002/zoo.20378

[CR165] Pereira TD, Tabris N, Matsliah A, Turner DM, Li J, Ravindranath S, Papadoyannis ES, Normand E, Deutsch DS, Wang ZY, McKenzie-Smith GC, Mitelut CC, Castro MD, D’Uva J, Kislin M, Sanes DH, Kocher SD, Wang S-H, Falkner AL, Murthy M (2022) SLEAP: A deep learning system for multi-animal pose tracking. Nat Methods 19(4):486–495. 10.1038/s41592-022-01426-1 35379947 10.1038/s41592-022-01426-1 PMC9007740

[CR166] Pineño O (2013) ArduiPod box: a low-cost and open-source Skinner box using an iPod Touch and an Arduino microcontroller. Behav Res Methods 46:196–205. 10.3758/s13428-013-0367-5 10.3758/s13428-013-0367-5 23813238

[CR167] Pitteri E, Mongillo P, Carnier P, Marinelli L, Huber L (2014) Part-Based and Configural Processing of Owner’s Face in Dogs. PLOS ONE 9(9):e108176. 10.1371/journal.pone.0108176 25251285 10.1371/journal.pone.0108176 PMC4177116

[CR168] Pongrácz P, Miklósi Á, Dóka A, Csányi V (2003) Successful application of video-projected human images for signalling to dogs. Ethology 109(10):809–821. 10.1046/j.0179-1613.2003.00923.x

[CR169] Pongrácz P, Ujvári V, Faragó T, Miklósi Á, Péter A (2017) Do you see what i see? The difference between dog and human visual perception may affect the outcome of experiments. Behav Processes 140:53–60. 10.1016/j.beproc.2017.04.002 28396145 10.1016/j.beproc.2017.04.002

[CR170] Potter RL, Weldon LJ, Shneiderman B (1988) Improving the accuracy of touch screens: An experimental evaluation of three strategies. Proc SIGCHI Conf Human Fact Comput Syst CHI ’88:27–32. 10.1145/57167.57171

[CR171] Prichard A, Chhibber R, Athanassiades K, Chiu V, Spivak M, Berns GS (2021) 2D or not 2D? An fMRI study of how dogs visually process objects. Anim Cogn 24(5):1143–1151. 10.1007/s10071-021-01506-3 33772693 10.1007/s10071-021-01506-3

[CR172] Rance S (2024) Manyfishes 1: A Standardized Test of Inhibitory Control in Fishes Using Big Team Science. Posters. https://digitalcommons.pittstate.edu/posters_2024/35

[CR173] Range F, Aust U, Steurer M, Huber L (2008) Visual categorization of natural stimuli by domestic dogs. Anim Cogn 11(2):339–347. 10.1007/s10071-007-0123-2 18026761 10.1007/s10071-007-0123-2

[CR174] Rayna T, Striukova L (2021) Assessing the effect of 3D printing technologies on entrepreneurship. Technol Forecast Soc Change 164:120483. 10.1016/j.techfore.2020.120483

[CR176] Renner E, Kean D, Atkinson M, Caldwell CA (2021) The use of individual, social, and animated cue information by capuchin monkeys and children in a touchscreen task. Sci Rep. 10.1038/s41598-020-80221-4 33441782 10.1038/s41598-020-80221-4 PMC7806602

[CR175] Renner E, Price EE, Subiaul F (2016) Sequential recall of meaningful and arbitrary sequences by orangutans and human children: does content matter? Anim Cogn 19(1):39–52. 10.1007/s10071-015-0911-z 26298671 10.1007/s10071-015-0911-z

[CR177] Reynolds CR, Altmann RA, Allen DN (2021) The Problem of Bias in Psychological Assessment. Mastering Modern Psychological Testing. Springer, Cham, pp 573–613. 10.1007/978-3-030-59455-8_15

[CR179] Rivas-Blanco D, Monteiro T, Virányi Z, Range F (2024) Going back to basics: Harlow’s learning set task with wolves and dogs. Learn Behav 52(4):315–329. 10.3758/s13420-024-00631-6 38780876 10.3758/s13420-024-00631-6 PMC11628440

[CR178] Rivas-Blanco D, Pohl I-M, Dale R, Heberlein MTE, Range F (2020) Wolves and dogs may rely on non-numerical cues in quantity discrimination tasks when given the choice. Front Psychol. 10.3389/fpsyg.2020.573317 33041945 10.3389/fpsyg.2020.573317 PMC7518719

[CR180] Romero-Ferrero F, Bergomi MG, Hinz RC, Heras FJH, De Polavieja GG (2019) idtracker.ai: tracking all individuals in small or large collectives of unmarked animals. Nat Methods 16(2):179–182. 10.1038/s41592-018-0295-5 30643215 10.1038/s41592-018-0295-5

[CR181] Rosenthal R (1994) Interpersonal expectancy effects: a 30-year perspective. Curr Dir Psychol Sci 3(6):176–179. 10.1111/1467-8721.ep10770698

[CR182] Rosnow RL, Rosenthal R (1976) The volunteer subject revisited. Aust J Psychol 28(2):97–108. 10.1080/00049537608255268

[CR183] Saad Saoud L, Sultan A, Elmezain M, Heshmat M, Seneviratne L, Hussain I (2024) Beyond observation: deep learning for animal behavior and ecological conservation. Ecol Inform 84:102893. 10.1016/j.ecoinf.2024.102893

[CR184] Sato Y, Sakai Y, Hirata S (2023) State-transition-free reinforcement learning in chimpanzees (*Pan troglodytes*). Learn Behav 51(4):413–427. 10.3758/s13420-023-00591-3 37369920 10.3758/s13420-023-00591-3

[CR185] Scheel B (2018) Designing digital enrichment for orangutans. Proc Fifth Int Conf Animal-Comput Int 1–11. 10.1145/3295598.3295603

[CR186] Schmidt FL, Hunter JE (1996) Measurement error in psychological research: lessons from 26 research scenarios. Psychol Methods 1(2):199–223. 10.1037/1082-989X.1.2.199

[CR187] Schmitt V (2018) Implementing new portable touchscreen-setups to enhance cognitive research and enrich zoo-housed animals. bioRxiv. 10.1101/316042

[CR188] Scholl BJ, Tremoulet PD (2000) Perceptual causality and animacy. Trends Cogn Sci 4(8):299–309. 10.1016/S1364-6613(00)01506-0 10904254 10.1016/s1364-6613(00)01506-0

[CR189] Schultz J, Frith CD (2022) Animacy and the prediction of behaviour. Neurosci Biobehav Rev 140:104766. 10.1016/j.neubiorev.2022.104766 35798127 10.1016/j.neubiorev.2022.104766

[CR190] Seitz BM, McCune K, MacPherson M, Bergeron L, Blaisdell AP, Logan CJ (2021) Using touchscreen equipped operant chambers to study animal cognition. Benefits, limitations, and advice. PLoS One 16(2):e0246446. 10.1371/journal.pone.0246446 33606723 10.1371/journal.pone.0246446 PMC7894864

[CR191] Shao Z, Yuan S, Jin Y, Wang Y (2024) Scholar’s career switch from academia to industry: mining and analysis from AMiner. Big Data Res 36:100441. 10.1016/j.bdr.2024.100441

[CR192] Sheldon EL, Hart CJ, Wilkinson A, Soulsbury C, Ratcliffe VF, Mills DS (2024) Working dogs in dynamic on-duty environments: the impact of dark adaptation, strobe lighting and acoustic distraction on task performance. PLoS One 19(2):e0295429. 10.1371/journal.pone.0295429 38330038 10.1371/journal.pone.0295429 PMC10852332

[CR193] Shneiderman B, Plaisant C (2005) Designing the user interface: Strategies for effective human-computer interaction, 4. ed. Pearson/Addison-Wesley

[CR194] Simonsohn U, Nelson LD, Simmons JP (2014) P-curve: a key to the file-drawer. J Exp Psychol Gen 143(2):534–547. 10.1037/a0033242 23855496 10.1037/a0033242

[CR195] Siniscalchi M, d’Ingeo S, Fornelli S, Quaranta A (2017) Are dogs red–green colour blind? R Soc Open Sci 4(11):170869. 10.1098/rsos.170869 29291080 10.1098/rsos.170869 PMC5717654

[CR196] Siniscalchi M, d’Ingeo S, Quaranta A (2023) Effect of attentional bias on the 3D rotated objects recognition ability of dogs. Animals 13(10):10. 10.3390/ani13101673 10.3390/ani13101673 PMC10215826 37238104

[CR197] Smith JG, Krichbaum S, Rogers B, Waggoner P, Katz JS, Lazarowski L (2025) Impact of variations in training schedules on dogs’ acquisition and retention of an odor detection task. Appl Anim Behav Sci 282:106474. 10.1016/j.applanim.2024.106474

[CR198] Somppi S, Törnqvist H, Hänninen L, Krause C, Vainio O (2012) Dogs do look at images: eye tracking in canine cognition research. Anim Cogn 15(2):163–174. 10.1007/s10071-011-0442-1 21861109 10.1007/s10071-011-0442-1

[CR199] Spanagel R (2022) Ten points to improve reproducibility and translation of animal research. Front Behav Neurosci. 10.3389/fnbeh.2022.869511 35530730 10.3389/fnbeh.2022.869511 PMC9070052

[CR200] Spetch ML, Cheng K, Mondloch MV (1992) Landmark use by pigeons in a touch-screen spatial search task. Anim Learn Behav 20(3):281–292. 10.3758/BF03213382

[CR201] Steurer MM, Aust U, Huber L (2012) The Vienna comparative cognition technology (VCCT): an innovative operant conditioning system for various species and experimental procedures. Behav Res Methods 44(4):909–918. 10.3758/s13428-012-0198-9 22437512 10.3758/s13428-012-0198-9

[CR202] Sun Y, Zhang J, Wang Q, Ni J (2025) RpiBeh offers a versatile open source solution for rodent behavior tracking and closed loop interventions. Sci Rep 15(1):29429. 10.1038/s41598-025-14693-7 40790325 10.1038/s41598-025-14693-7 PMC12340062

[CR203] Tanaka T, Watanabe T, Eguchi Y, Yoshimoto T (2000) Color discrimination in dogs. Nihon Chikusan Gakkaiho 71(3):300–304. 10.2508/chikusan.71.300

[CR204] Tapp PD, Siwak CT, Estrada J, Head E, Muggenburg BA, Cotman CW, Milgram NW (2003) Size and reversal learning in the Beagle dog as a measure of executive function and inhibitory control in aging. Learn Mem 10(1):64–73. 10.1101/lm.54403 12551965 10.1101/lm.54403 PMC196651

[CR205] Toegel F, Toegel C, Perone M (2021) Design and evaluation of a touchscreen apparatus for operant research with pigeons. J Exp Anal Behav 116(2):249–264. 10.1002/jeab.707 34236081 10.1002/jeab.707 PMC8882370

[CR206] Torrisi B, Pernagallo G (2020) Investigating the relationship between job satisfaction and academic brain drain: the Italian case. Scientometrics 124(2):925–952. 10.1007/s11192-020-03509-2

[CR207] Truax J, Vonk J (2023) Silence is Golden: auditory preferences in zoo-housed gorillas. J Appl Anim Welfare Sci 26(3):404–419. 10.1080/10888705.2021.1968400 10.1080/10888705.2021.1968400 34428085

[CR208] Tuyttens FAM, de Graaf S, Heerkens JLT, Jacobs L, Nalon E, Ott S, Stadig L, Van Laer E, Ampe B (2014) Observer bias in animal behaviour research: can we believe what we score, if we score what we believe? Anim Behav 90:273–280. 10.1016/j.anbehav.2014.02.007

[CR209] Uexküll J (2001) An introduction to Umwelt. 2001 134:107–110. 10.1515/semi.2001.017

[CR210] Uexküll J, Kriszat G (2013) Streifzüge durch die umwelten von tieren und menschen ein bilderbuch unsichtbarer welten. Springer-Verlag

[CR211] Vernouillet AAA, Stiles LR, McCausland J, Kelly DM (2018) Individual performance across motoric self-regulation tasks are not correlated for pet dogs. Learn Behav 46(4):522–536. 10.3758/s13420-018-0354-x 30251102 10.3758/s13420-018-0354-x

[CR212] Völter CJ, Karl S, Huber L (2020) Dogs accurately track a moving object on a screen and anticipate its destination. Sci Rep 10(1):19832. 10.1038/s41598-020-72506-5 33199751 10.1038/s41598-020-72506-5 PMC7670446

[CR213] Vonk J (2024) Prosocial or photo preferences? Gorillas’ prosocial choices using a touchscreen. Am J Primatol 86(5):e23612. 10.1002/ajp.23612 38425016 10.1002/ajp.23612

[CR214] Vonk J, Truax J, McGuire MC (2022) A food for all seasons: stability of food preferences in gorillas across testing methods and seasons. Animals 12(6):685. 10.3390/ani12060685 35327082 10.3390/ani12060685 PMC8944577

[CR227] Waldin, N ., Waldner, M ., & Viola, I. (2017). Flicker Observer Effect: Guiding Attention Through High Frequency Flicker in Images. Computer Graphics Forum, 36(2), 467–476. 10.1111/cgf.13141

[CR215] Wallis LJ, Virányi Z, Müller CA, Serisier S, Huber L, Range F (2016) Aging effects on discrimination learning, logical reasoning and memory in pet dogs. AGE 38(1):6. 10.1007/s11357-015-9866-x 26728398 10.1007/s11357-015-9866-x PMC5005891

[CR216] Wark JD, Cronin KA, Niemann T, Shender MA, Horrigan A, Kao A, Ross MR (2019) Monitoring the behavior and habitat use of animals to enhance welfare using the ZooMonitor app. Anim Behav Cogn 6(3):158–167. 10.26451/abc.06.03.01.2019

[CR217] Wasserman EA, Nagasaka Y, Castro L, Brzykcy SJ (2013) Pigeons learn virtual patterned-string problems in a computerized touch screen environment. Anim Cogn 16(5):737–753. 10.1007/s10071-013-0608-0 23397181 10.1007/s10071-013-0608-0

[CR218] Webster MM, Rutz C (2020) How STRANGE are your study animals? Nature 582(7812):337–340. 10.1038/d41586-020-01751-5 32541916 10.1038/d41586-020-01751-5

[CR219] Wigdor D, Wixon D (2011) Brave NUI World: Designing Natural User Interfaces for Touch and Gesture. Elsevier

[CR220] Wilson E, Ramage FJ, Wever KE, Sena E, Macleod MR, Currie GL (2023) Designing, conducting, and reporting reproducible animal experiments. J Endoc 25*8*. 10.1530/joe-22-0330 10.1530/JOE-22-0330 PMC10304908 37074416

[CR221] Wobbrock JO, Kane SK, Gajos KZ, Harada S, Froehlich J (2011) Ability-based design: concept, principles and examples. ACM Trans Access Comput 3(3):1–27. 10.1145/1952383.1952384

[CR222] Woolston C (2020) Postdoc survey reveals disenchantment with working life. Nature 587(7834):505–508. 10.1038/d41586-020-03191-7 33208965 10.1038/d41586-020-03191-7

[CR223] Ye S, Lauer J, Zhou M, Mathis A, Mathis M (2023) AmadeusGPT: a natural language interface for interactive animal behavioral analysis. Adv Neural Inf Process Syst 36:6297–6329

[CR224] Yin S, Fernandez EJ, Pagan S, Richardson SL, Snyder G (2008) Efficacy of a remote-controlled, positive-reinforcement, dog-training system for modifying problem behaviors exhibited when people arrive at the door. Appl Anim Behav Sci 113(1–3):123–138. 10.1016/j.applanim.2007.11.001

[CR225] Zeagler C, Gilliland S, Freil L, Starner T, Jackson M (2014) Going to the dogs: Towards an interactive touchscreen interface for working dogs. Proc 27th Annual ACM Sympos User Int Softw Technol 497–507. 10.1145/2642918.2647364

[CR226] Zeagler C, Zuerndorfer J, Lau A, Freil L, Gilliland S, Starner T, Jackson MM (2016) Canine computer interaction: Towards designing a touchscreen interface for working dogs. Proc Third Int Conf Animal-Comput Int 1–5. 10.1145/2995257.2995384

